# Genotypic variation in yield, physiological traits, and drought tolerance of quinoa (*Chenopodium quinoa* Willd.) under arid conditions

**DOI:** 10.3389/fpls.2025.1679444

**Published:** 2025-11-18

**Authors:** Magdi A. A. Mousa, Khalid A. Asiry, Adel D. Al-qurashi, Najeeb M. Almasoudi, Mohammed I. Elsayed

**Affiliations:** Department of Agriculture, Faculty of Environment Sciences, King Abdulaziz University, Jeddah, Saudi Arabia

**Keywords:** quinoa, drought tolerance, irrigation regimes, seed yield, chlorophyll, PCA, arid agriculture, genotypic variation

## Abstract

Water scarcity is a major constraint to agricultural productivity in arid and semi-arid regions, underscoring the need for crops with high water-use efficiency and resilience to drought. This study evaluated 21 quinoa (Chenopodium quinoa Willd.) accessions under three drip-irrigation regimes—W1 (15 min), W2 (10 min), and W3 (5 min)—applied twice daily in a split-plot design with three replications in the arid conditions of western Saudi Arabia. Significant effects of irrigation, genotype, and their interaction were detected for most phenological, morphological, physiological, and yield traits. Among the evaluated accessions, CHEN-195, CHEN-316, and CHEN-140 consistently outperformed others, producing the highest seed yields per plant (up to 13.58 g) and per hectare (2400.2 kg ha^-1^), with greater 1000-seed weight and stable chlorophyll a and b contents across growth stages. Principal component analysis explained 70.7 % of the total variation, identifying yield, plant height, and drought tolerance indices as the principal contributors to genotype differentiation. Heatmap clustering confirmed distinct performance groups, reinforcing the robustness of multivariate classification in discriminating drought-adapted genotypes. The combined results highlight considerable genetic variability in quinoa response to irrigation levels and identify promising accessions with superior adaptation and resource-use efficiency. Overall, this study supports the integration of quinoa into dryland farming systems and provides a foundation for breeding programs targeting enhanced drought tolerance and sustainable production under water-limited conditions.

## Graphical Abstract

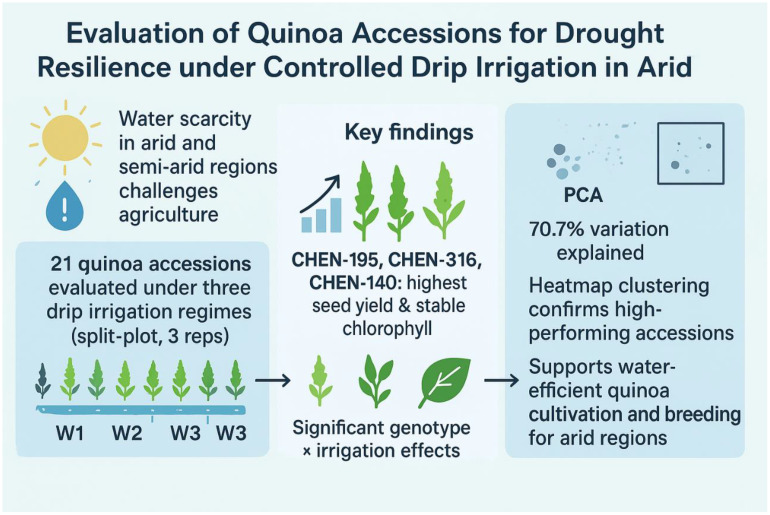

## Introduction

Globally, over 70% of total water resources are allocated to agricultural irrigation (Frimpong et al., 2023). Escalating freshwater scarcity necessitates the development of efficient, crop-specific irrigation strategies to enhance water-use efficiency and maintain agricultural productivity, especially in arid and semi-arid regions (Bazile et al., 2016). The combined effects of drought and rising temperatures represent major abiotic stressors, reducing plant morphological and physiological performance by lowering leaf water potential, sap flow, stomatal conductance, and xylem function, ultimately diminishing crop productivity (Bhusal et al., 2019; Marengo et al., 2021). The Middle East and North Africa (MENA) region, including Saudi Arabia, is among the most water-stressed areas globally. According to the World Bank, countries with annual renewable water resources below 1,700 m³ per capita are classified as water-stressed (Benlhabib et al., 2016; Nanduri et al., 2019; Al-Naggar et al., 2020; Hejazi et al., 2023). According to FAO data, per capita renewable water resources in MENA have declined extremely from about 1,752 m³ in 1970 to roughly 530 m³ by 2020 indicating that many countries in the region now fall well below the water-stress and scarcity thresholds (Lahham et al., 2022). Saudi Arabia receives only 50–150 mm of rainfall annually, with erratic precipitation patterns (Almazroui, 2020). In Saudi Arabia, agriculture accounts for approximately 78% of total national water demand (11.4 out of ~14.3 BCM/year in 2021), while irrigation efficiency remains low around 50% compared to global best practices of ~85% (Al-Zahrani and Khan, 2023). Although Saudi Arabia leads the world in seawater desalination, growing pressure on aquifers and limited natural recharge from rainfall continue to jeopardize long-term water security (Abdella et al., 2024). To address these constraints, the country has adopted policies to reduce water-intensive crop cultivation, improve irrigation technologies, and explore alternative cropping systems including halophyte farming (Multsch et al., 2017). One such promising halophytic crop is quinoa (Chenopodium quinoa Willd.), a highly adaptable pseudocereal originating from the Andean region. It thrives in marginal environments, tolerating drought, salinity, and frost (Alandia et al., 2020; González et al., 2021). Quinoa is a facultative halophyte with a C₃ photosynthetic pathway and has shown strong agronomic performance under a wide range of agroecological conditions (Afzal et al., 2022). Its grains are nutritionally rich, providing essential proteins, amino acids, fatty acids, vitamins, and minerals, making it valuable for food and nutritional security (Repo-Carrasco et al., 2003; Stikic et al., 2012). In addition, quinoa has been used as a food crop, forage, cover crop, and phytoremediation agent (Hinojosa et al., 2018). The life cycle of quinoa comprises both vegetative and reproductive stages influenced by photoperiod and temperature, contributing to its broad genetic diversity and adaptive capacity (Afzal et al., 2022; Alandia et al., 2021). Genotypes vary in flowering and maturity timing, ranging from 109 to 182 days (Hafeez et al., 2022). Under drought or rainfed conditions, quinoa exhibits various adaptive traits, such as increased root proliferation, reduced shoot biomass, early flowering, and stomatal closure, enabling survival under limited water availability (Jacobsen et al., 2009; Zurita Silva et al., 2015a; b; Choukr-Allah et al., 2016). Its antioxidant-rich profile, including flavonoids and anthocyanins, provides added protection against abiotic stresses (Saad-Allah and Youssef, 2018; Wang et al., 2020; Razzaghi et al., 2020). Antioxidants help drought tolerance by scavenging reactive oxygen species (ROS) that accumulate under water deficit, thereby protecting cellular structures such as membranes, proteins, and DNA from oxidative damage. Additionally, they stabilize photosynthetic pigments and maintain enzyme activities, supporting continued growth and metabolic function under stress conditions (Dabravolski et al., 2023). Recent advancements in quinoa phenotyping and digital agriculture have enhanced our understanding of its stress response. UAV-based approaches, such as those using SPAD-derived chlorophyll indices and machine learning, have proven effective for large-scale diversity evaluations (Jiang et al., 2021). Standardized phenotyping protocols have also been established to ensure data comparability and reproducibility across research sites and environments (Stanschewski et al., 2021; Alghanem et al., 2021). These innovations are particularly valuable for screening accessions for drought tolerance traits under field conditions. In Saudi Arabia, quinoa is increasingly regarded as a viable crop for ensuring food and water security. Its high tolerance to salinity and arid climates aligns well with the Kingdom’s strategy to diversify cropping systems and improve sustainability in agriculture (Alrwis et al., 2021; Shams, 2022). Field trials and research initiatives have demonstrated quinoa’s strong adaptability and yield potential under Saudi conditions, particularly when grown with efficient irrigation management (Hinojosa et al., 2019; El-Harty et al., 2021). In this context, quinoa (*Chenopodium quinoa* Willd.) has emerged as a resilient crop with considerable potential for cultivation under arid and semi-arid environments due to its tolerance to drought and salinity. However, genotype × environment interactions remain poorly understood, particularly under the specific water-limited conditions of the western region of Saudi Arabia, where agricultural productivity is increasingly constrained by declining water resources. Therefore, this study aimed to evaluate the agronomic performance, yield stability, and physiological responses of diverse quinoa accessions under contrasting irrigation regimes. By linking global concerns of water scarcity with localized field evaluation, this research provides insights into the potential of quinoa as a strategic crop to enhance food security in water-scarce regions.

## Materials and methods

### Investigation site

This study was conducted at the Agricultural Research Station of King Abdulaziz University in Hada Al-Sham, situated in the western region of Saudi Arabia (21.7961° N, 39.7256° E). The site experiences an arid climate, characterized by low and highly variable annual rainfall (ranging from 50 to 100 mm), elevated temperatures, and sandy-loam soil with limited water-holding capacity and low organic matter. The soil pH measured 7.8, and electrical conductivity (EC) was 1.79 dS/m, indicating moderately saline conditions. Detailed soil characteristics are presented in **Table 1**. These environmental features are typical of dryland farming systems across the Arabian Peninsula (Odokonyero et al., 2024), making the site ideal for evaluating crop responses under water-limited conditions.

**Table 1:** Soil physical and chemical properties of the KAU Agriculture Research Station at Hada Al Sham, Al-Jamoom, Saudi Arabia.

pH (unite)	EC (ds/m)	Sandy loam soil particle size (%)			Organic matter (%)		Organic carbon (%)		Available macro nutrients (mg/kg)		
		Sand	Silt	Clay					N	P	K
7.83	1.79	84.21	14.05	1.74	0.453		0.500		0.215	0.070	0.781
Key elements (mg/kg)											
Cr	Pb	Ni	Cd	Mn	Fe	Ca	Mg	Cu	Zn	Na	
0.11	4.21	0.52	0.06	144.44	239.40	1.38	1.15	4.78	32.98	0.14	

### Plant materials

A total of 21 quinoa (Chenopodium quinoa Willd.) accessions were evaluated in this study. The accessions were generously provided by Prof. Mark Tester from King Abdullah University of Science and Technology (KAUST). The accessions were sourced from international and regional germplasm collections, including varieties adapted to a wide range of environments. These genotypes represent a diverse range of geographical origins and genetic backgrounds, and they differ in morphological traits such as panicle color and growth habit. This genetic diversity offered a robust framework for assessing performance under contrasting irrigation regimes. Detailed descriptions of the accessions, including their origins and panicle coloration, are listed in **Table 2** (Jiang et al., 2022).

**Table 2:** Origin, source, and panicle color of the quinoa (Chenopodium quinoa Willd.) accessions evaluated under different irrigation regimes in arid conditions.

Accession code	Origin/source	Panicle color	Reference
CHEN-195	Bolivia	Red	KAUST Germplasm Collection, 2023
CHEN-316	Peru	Yellow	Hinojosa et al., 2018
CHEN-140	Chile	Pink	USDA GRIN, 2020
PI-614919	Bolivia(Oruro)	Green	USDA GRIN, 2020
D-12361	Ecuador	Orange	Bazile et al., 2016
BO-63	Bolivia	Red	Hinojosa et al., 2018
BO-40	Bolivia	Red	Bazile et al., 2016
BO-60	Bolivia	Brown	USDA GRIN, 2020
CICA-17	Peru	Cream	Razzaghi et al., 2020
MHW-1	Morocco	Yellow	Fghire et al., 2015
PI-614935	Bolivia(Oruro)	Green	USDA GRIN, 2020
D-12075	Ecuador	Pink	Bazile et al., 2016
AMES-13721	Argentina	Purple	USDA GRIN, 2020
Regalona	Bolivia	Yellow	USDA GRIN, 2020
D-11891	Chile	Orange	KAUST Germplasm Collection, 2023
BO-11	Peru	Green	Hinojosa et al., 2018
PI-634919	Chile	Red	Fghire et al., 2015
CHEN-389	Argentina	Yellow	USDA GRIN, 2020
D-9973	Peru	Pink	Razzaghi et al., 2020
D-12085	Bolivia	Brown	Hinojosa et al., 2018
Ames-13740	Chile	Cream	Bazile et al., 2016

### Experimental design and layout

The experiment followed a split-plot design with three replications. The main plots were assigned to three irrigation regimes, and the sub-plots to the 21 quinoa accessions, which were randomly arranged within each replication. Each subplot measured 1 m × 1 m, with rows spaced 30 cm apart and 10 cm between planting hills. Each hill was initially seeded with four seeds, and thinning was performed 15 days after sowing to retain one healthy plant per hill, ensuring uniform plant density across treatments. The three irrigation regimes were designed to simulate different levels of water availability: W1, 15 minutes twice daily (full irrigation, ~24 L/m^2^/day); W2, 10 minutes twice daily (moderate irrigation, ~16 L/m^2^/day); and W3, 5 minutes twice daily (deficit irrigation, ~8 L/m^2^/day). Irrigation treatments began after thinning and were maintained consistently throughout the growth cycle. All plots received standardized agronomic management, including fertilization, weeding, and pest control, based on quinoa production guidelines (Taaime et al., 2023).

### Plant growth conditions and data collection

To ensure consistent water application, a pressure-compensated drip irrigation system (Rain Bird LD-06-12-1000) was installed. This system delivered water at a rate of 4 L/h per emitter, with emitters spaced 30 cm apart along the laterals and the drip lines spaced at 25cm. Irrigation was applied twice daily, at 7:00 AM and 5:00 PM, in accordance with the treatment durations. Fertigation was applied weekly via the same system, using a balanced N:P:K fertilizer (20:20:20) during the vegetative stage, and a reproductive-stage formula (10:10:40) during flowering and seed development. This helped ensure uniform nutrient availability across treatments. A wide range of plant parameters were measured: Phenological traits: Days to 50% flowering and Days to seed set; Growth and yield-related traits (at harvest): Plant height (cm), Shoot dry weight (g), Root dry weight (g), Panicle dry weight (g), Seed yield per plant (g), Estimated seed yield per hectare (kg ha^-1^) and 1000-seed weight (g); Physiological traits (measured using samples of 5 representative plants/subplot): Chlorophyll a and b contents (mg/100 g fresh weight) were measured at 30, 60, and 90 days after sowing. SPAD readings were taken from the uppermost fully expanded leaves of each plant. Pigments were extracted using 80% acetone and quantified spectrophotometrically following the method of Lichtenthaler and Wellburn (1983); Stress tolerance indices: Yield Tolerance Index (YTI) and Drought Tolerance Index (DTI). These indexes were calculated based on yield performance under stress and non-stress conditions, following the approach of Fernandez (1992).

### Data analysis

All recorded data were analyzed using a split-plot analysis of variance (ANOVA) to account for the experimental design. Irrigation regimes were considered as the main plot factor, while genotypes were assigned to subplots, with replicates treated as random effects. Mean comparisons were performed using the least significant difference (LSD) test at the 5% probability level (P < 0.05). Statistical analyses were conducted using SAS software (version 9.4, SAS Institute Inc., Cary, NC, USA). To further explore trait associations and genotype performance: Principal Component Analysis (PCA) was conducted to identify the major traits contributing to variability across genotypes and treatments. PCA biplots were generated using the FactoMineR and factoextra packages in R (Husson et al., 2017). A hierarchical clustered heatmap was created to visualize phenotypic groupings among the quinoa accessions. Standardized trait values were used, and clustering was based on Euclidean distance and Ward’s linkage method, implemented via the heatmap package in R. These multivariate analyses helped distinguish high-performing genotypes and traits associated with drought resilience and productivity under arid conditions.

## Results and discussion

This study evaluated the performance of 21 quinoa accessions under three water regimes: W1 (15 min twice/day), W2 (10 min twice/day), and W3 (5 min twice/day) in arid conditions typical of Saudi Arabia. The following discussion presents a detailed and integrated interpretation of morphological and yield traits, focusing on genotype–irrigation interactions and highlighting traits that contribute to quinoa resilience and

### Days to 50% flowering

Days to 50% flowering was significantly influenced by both genotype and irrigation regime (**Table 3**), although their interaction was not significant, indicating consistent genotypic behavior across water treatments. Mean flowering time was delayed with increased irrigation: W1 (15 min twice/day) recorded the latest flowering (42.41 days), followed by W2 (38.21 days), while the earliest occurred under W3 (32.35 days). Genotypes ‘Regalona’ and ‘CICA-17’ exhibited the longest flowering duration, while ‘CHEN-140’, ‘CHEN-195’, and ‘CHEN-316’ flowered earliest, suggesting potential drought escape ability. The absence of a significant interaction suggests that flowering time is largely under genetic control, and thus stable across environments. These results support prior work by Peterson and Murphy (2015) and Bazile et al. (2016), which found that high soil moisture prolongs vegetative stages and delays reproductive development.

**Table 3:** Days to 50% flowering of 21 quinoa accessions under different irrigation regimes in arid conditions.

Accessions	Water level			Mean
	W1	W2	W3	
PI-614935	33.000 q-w	38.667 f-o	44.000 a-e	38.556 abc
PI-614919	34.000 o-v	37.667 g-q	42.000 a-h	37.889 bc
D-12361	35.333 l-u	38.000 f-p	44.333 abcd	39.222 ab
D-12075	35.667 k-u	39.333 e-n	43.667 a-e	39.556 ab
BO-63	31.333 t-x	35.333 l-u	42.000 a-h	36.222 cd
CHEN-316	28.333 wx	35.000 m-u	37.667 g-q	33.667 def
Regalona	34.667 n-u	41.667 a-i	45.667 a	40.667 a
CHEN-140	28.333 wx	33.667 p-v	37.333 h-r	33.111 f
BO-60	32.000 s-x	36.333 j-s	41.000 a-j	36.444 c
D-11891	36.000 k-t	40.333 b-k	44.667 abc	40.333 ab
MHW-1	32.667 r-w	39.667 d-m	45.333 a	39.222 ab
BO-11	31.000 uvwx	37.000 i-r	40.000 c-l	36.000 cde
CHEN-195	27.667 x	34.667 n-u	38.000 f-p	33.444 ef
PI-634919	34.000 o-v	40.333 b-k	43.667 a-e	39.333 ab
CHEN-389	28.333 wx	35.000 m-u	37.333 h-r	33.556 def
BO-40	32.000 s-x	38.000 f-p	43.667 a-e	37.889 bc
D-9973	35.333 l-u	40.333 b-k	44.000 a-e	39.889 ab
D-12085	34.667 n-u	40.000 c-l	44.333 abcd	39.667 ab
Ames-13721	29.667 vwx	39.667 d-m	44.333 abcd	37.889 bc
Ames-13740	32.000 s-x	39.333 e-n	42.66 a-f	38.000 abc
CICA-17	33.333 p-v	42.333 a-g	45.000 ab	40.222 ab
Mean	32.349 c	38.206 b	42.413 a	

Means having different alphabets indicates are statistically different at the 5% significance level, as determined by LSD test (P < 0.05); LSD for Water Level= 1.0455; LSD for Accessions = 2.7662; LSD for Water Level x Accessions= 4.7913.

### Days to seed set

Significant variation in days to seed set was observed across genotypes, irrigation levels, and their interaction. Early seed set was favored under reduced irrigation (W3), while prolonged durations were associated with W1. ‘CHEN-140’ showed the shortest seed set interval, especially under W3, while ‘Regalona’ exhibited the longest duration (**Table 4**). The mean days to seed set followed the trend W1 > W2 > W3, emphasizing the role of water availability in prolonging vegetative phases. The significant interaction between genotype and irrigation highlights differential adaptive strategies, where early seed setting genotypes may exhibit drought escape potential (**Table 4**). These findings align with reports by Adams et al. (2020) and Jacobsen et al. (2003) on phenological plasticity in quinoa.

**Table 4:** Days to seed set of 21 quinoa accessions under different irrigation regimes in arid conditions.

Accessions	Water level			Mean
	W1	W2	W3	
PI-614935	48.000 m-q	53.333 d-k	57.667 a-e	53.000 bc
PI-614919	47.667 nopq	52.667 f-m	55.667 b-g	52.000 c
D-12361	49.667 i-q	53.000 e-l	57.333 a-f	53.333 bc
D-12075	50.000 i-p	54.333 b-i	57.333 a-f	53.889 bc
BO-63	46.667 opqr	52.667 f-m	55.333 b-h	51.556 c
CHEN-316	40.333 t	46.333 opqr	49.333 j-q	45.333 d
Regalona	52.000 g-n	57.667 a-e	61.667 a	57.111 a
CHEN-140	40.333 t	45.000 qrst	8.667 k-q	44.667 d
BO-60	46.333 opqr	53.333 d-k	56.000 b-g	51.889 c
D-11891	50.667 h-o	53.000 e-l	58.000 abcd	53.889 bc
MHW-1	48.333 l-q	57.000 a-f	61.667 a	55.667 ab
BO-11	47.000 opqr	53.333 d-k	54.333 b-i	51.556 c
CHEN-195	40.667 st	46.667 opqr	48.333 l-q	45.222 d
PI-634919	42.667 rst	55.333 b-h	57.667 a-e	51.889 c
CHEN-389	40.333 t	45.667 pqr	48.000 m-q	44.667 d
BO-40	45.333 pqrs	52.667 f-m	57.000 a-f	51.667 c
D-9973	49.333 j-q	53.667 c-j	57.000 a-f	53.333 bc
D-12085	50.667 h-o	53.333 d-k	57.333 a-f	53.778 bc
Ames-13721	46.667 opqr	55.000 b-h	58.667 ab	53.444 bc
Ames-13740	45.667 pqr	54.000 b-j	57.667 abcde	52.444 c
CICA-17	50.667 h-o	58.333 abc	61.000 a	56.667 a
Mean	46.619 c	52.683 b	55.984 a	

Means having different alphabets indicates are statistically different at the 5% significance level, as determined by LSD test (P < 0.05). LSD for Water Level= 1.0334; LSD for Accessions = 2.7340; LSD for Water Level x Accessions= 4.7354.

### Plant height (cm)

Plant height varied significantly among irrigation regimes, genotypes, and their interaction. The tallest plants were observed under W1, with ‘CHEN-140’, ‘CHEN-316’, and ‘CHEN-195’ attaining heights above 115 cm. The shortest plant height was recorded in ‘AMES-13721’ under W3 (63. 25 cm), suggesting strong negative effects of water stress on vegetative growth. Overall, mean plant height declined from W1 (99. 91 cm) to W3 (79.48 cm), underscoring the importance of sufficient irrigation for optimal height development (**Table 3**). Genotype × irrigation interaction showed that accessions like ‘CHEN-140’ and ‘CHEN-316’ responded particularly well to increased water, suggesting suitability for irrigated environments (**Table 5**). These trends corroborate the results of AbdElgalil et al. (2023) and Razzaghi et al. (2020), who found that plant height in quinoa is sensitive to water deficits and serves as an indicator of drought stress response. (**Table 5**)

**Table 5:** Plant height (cm) of 21 quinoa accessions under different Irrigation Regimes in arid conditions.

Accessions	Water level			Mean
	W1	W2	W3	
PI-614935	72.75 X-d	89.67 I-P	104.75 CDE	89.06 B
PI-614919	75.08 U-c	73.33 W-c	103.17 C-G	83.86 BCD
D-12361	72.25 Z-d	79.33 Q-b	86.75 L-R	79.44 DE
D-12075	78.17 R-c	82.42 P-X	103.92 CDEF	88.17 BC
BO-63	83.08 O-V	85.58 M-R	96.58 E-K	88.42 BC
CHEN-316	98.25 C-I	103.42 C-G	115.42 AB	105.69 A
Regalona	84.00 N-U	69.58 cd	84.08 N-U	79.22 DE
CHEN-140	96.83 D-J	106.42 BCD	123.50 A	108.92 A
BO-60	81.25 P-a	88.58 I-Q	95.67 E-L	88.50 BC
D-11891	82.67 P-W	79.50 Q-b	102.00 C-H	88.06 BC
MHW-1	71.17 bcd	82.25 P-Y	97.00 D-J	83.47 BCD
BO-11	75.25 T-c	84.83 M-T	101.58 C-H	87.22 BC
CHEN-195	94.08 G-M	106.83 BC	115.75 AB	105.56 A
PI-634919	81.75 P-Z	83.25 O-V	93.00 H-N	86.00 BC
CHEN-389	94.42 F-M	104.33 CDE	116.58 A	105.11 A
BO-40	71.92 abcd	75.75 S-c	104.25 CDE	83.97 BCD
D-9973	72.67 Y-d	88.25 J-Q	92.75 H-O	84.56 BCD
D-12085	75.25 T-c	84.92 M-T	89.67 I-P	83.28 CD
Ames-13721	63.25 d	79.33 Q-b	85.08 M-S	75.89 E
Ames-13740	70.17 bcd	81.33 P-a	87.08 K-R	79.53 DE
CICA-17	75.00 U-c	74.17 V-c	99.58 C-H	82.92 CD
Mean	79.488 c	85.861 b	99.913 a	

Means having different alphabets indicates are statistically different at the 5% significance level, as determined by LSD test (P < 0.05). LSD for Water Level= 1.0720; LSD for Accessions = 5.6138; LSD for Water Level x Accessions= 9.7233.

### Shoot, panicle, and root dry weight (g)

Shoot, panicle, and root dry weights were all significantly influenced by irrigation regime, genotype, and their interaction (**Tables 6**, **7**, **8**). Across traits, the general trend was W1 > W2 > W3, reflecting the positive effect of higher water availability on biomass accumulation. However, notable genotypic differences emerged. Accessions ‘CHEN-140’ and ‘CHEN-316’ consistently recorded the highest shoot (89.67 and 88.67 g, respectively) and panicle dry weights (>47 g) under W1, indicating superior vegetative vigor and reproductive investment under optimal irrigation. In contrast, ‘AMES-13721’ and ‘PI-614919’ were among the lowest performers under W3 across all three biomass traits. Root biomass also followed the same irrigation trend, with ‘CHEN-316’ and ‘CHEN-140’ producing the highest values under W1, while maintaining relatively higher root dry weight under W3. This suggests deeper or more efficient root systems, a trait associated with drought resilience (Geerts et al., 2008). The consistency of CHEN accessions in maintaining superior shoot, panicle, and root dry weights under stress aligns with previous findings (Razzaghi et al., 2020; Bazile et al., 2016), highlighting their adaptability and potential as parents in breeding for drought-prone environments.

**Table 6:** Root dry weight (g) of 21 quinoa accessions under different irrigation regimes in arid conditions.

Accessions	Water level			Mean
	W1	W2	W3	
PI-614935	20.750 defg	17.917 f-j	17.667 g-k	18.778 bc
PI-614919	19.167 d-h	15.667 ijkl	18.667 d-i	17.833 cd
D-12361	20.833 defg	17.667 g-k	20.583 defg	19.694 bc
D-12075	22.000 cd	17.917 f-j	20.917 defg	20.278 b
BO-63	21.750 cde	14.333 kl	19.417 d-h	18.500 bc
CHEN-316	28.833 a	25.917 ab	25.583 ab	26.778 a
Regalona	20.083 d-h	18.167 f-j	20.000 d-h	19.417 bc
CHEN-140	28.000 ab	24.833 bc	25.917 ab	26.250 a
BO-60	20.083 d-h	19.000 d-i	14.333 kl	17.806 cd
D-11891	18.833 d-i	18.417 e-j	19.250 d-h	18.833 bc
MHW-1	19.750 d-h	18.500 e-j	18.583 d-ij	18.944 bc
BO-11	19.083 d-i	19.917 d-h	19.667 d-h	19.556 bc
CHEN-195	27.750 ab	24.750 bc	26.000 ab	26.167 a
PI-634919	20.000 d-h	14.000 l	21.167 def	18.389 bc
CHEN-389	27.333 ab	24.917 bc	24.833 bc	25.694 a
BO-40	19.417 d-h	19.833 d-h	16.917 h-l	18.722 bc
D-9973	20.250 d-h	19.583 d-h	18.167 f-j	19.333 bc
D-12085	19.583 d-h	15.167 jkl	14.333 kl	16.361 d
Ames-13721	19.833 d-h	18.250 f-j	19.750 d-h	19.278 bc
Ames-13740	19.500 d-h	17.000 h-l	21.083 defg	19.194 bc
CICA-17	20.083 d-h	19.333 d-h	17.750 f-k	19.056 bc
Mean	21.567 a	19.099 c	20.028 b	

Means having different alphabets indicates are statistically different at the 5% significance level, as determined by LSD test (P < 0.05). LSD for Water Level= 0.7607; LSD for Accessions = 2.0126; LSD for Water Level x Accessions= 3.4860.

**Table 7:** Panicle dry weight (g) of 21 quinoa accessions under different irrigation regimes in arid conditions.

Accessions	Water level			Mean
	W1	W2	W3	
PI-614935	58.667 Q-a	63.410 J-N	66.000 HIJ	62.692 DE
PI-614919	55.330 abcd	54.733 bcd	61.873 K-R	57.312 J
D-12361	57.663 T-c	57.943 T-c	59.637 O-X	58.414 HIJ
D-12075	61.357 K-T	58.073 S-c	67.877 GHI	62.436 DE
BO-63	58.797 O-a	55.873 Y-d	60.803 M-V	58.491 HIJ
CHEN-316	74.477 CDE	71.587 EFG	77.670 ABC	74.578 B
Regalona	62.510 J-O	55.803 Z-d	60.410 M-W	59.574 FGHI
CHEN-140	78.260 AB	74.983 A-E	78.667 A	77.303 A
BO-60	61.260 K-U	60.947 L-U	58.377 R-b	60.194 FGH
D-11891	61.267 K-U	60.000 N-W	69.093 FGH	63.453 D
MHW-1	58.177 R-c	62.207 K-Q	62.360 J-Q	60.914 EFG
BO-11	60.017 N-W	60.367 M-W	64.527 IJKL	61.637 DEF
CHEN-195	71.917 EF	70.150 FG	74.817 BCDE	72.294 C
PI-634919	57.607 U-d	56.063 X-d	59.767 N-X	57.812 IJ
CHEN-389	69.580 FGH	72.797 DEF	76.407 ABCD	72.928 BC
BO-40	53.923 d	56.143 X-d	62.393 J-P	57.487 IJ
D-9973	56.100 X-d	60.833 L-V	59.370 O-Z	58.768 HIJ
D-12085	61.673 K-S	56.857 W-d	57.817 T-c	58.782 GHIJ
Ames-13721	54.607 cd	57.737 T-c	59.543 O-Y	57.296 J
Ames-13740	56.160 X-d	57.917 T-c	63.837 JKLM	59.304 GHIJ
CICA-17	57.133 V-d	58.687 P-a	64.960 IJK	60.260 FGH
Mean	61.261 b	61.100 b	65.057 a	

Means having different alphabets indicates are statistically different at the 5% significance level, as determined by LSD test (P < 0.05). LSD for Water Level= 0.8108; LSD for Accessions = 2.1452; LSD for Water Level x Accessions= 3.7156.

**Table 8:** Shoot dry weight (g) of 21 quinoa accessions under different irrigation regimes in arid conditions (**Table 5**).

Accessions	Water level			Mean
	W1	W2	W3	
PI-614935	62.083 V-c	69.263 J-N	74.247 HI	68.531 CDE
PI-614919	58.747 cd	59.750 bcd	69.090 KLMN	62.529 JK
D-12361	62.330 U-c	64.097 O-b	67.130 L-S	64.519 HIJK
D-12075	66.360 N-V	63.673 Q-b	76.600 GH	68.878 CD
BO-63	64.160 O-a	61.757 W-c	68.833 LMN	64.917 GHIJ
CHEN-316	81.930 DE	80.893 DEFG	88.417 AB	83.747 B
Regalona	67.053 M-T	60.840 Z-d	66.510 N-U	64.801 HIJK
CHEN-140	86.577 ABC	85.143 BCD	89.667 A	87.129 A
BO-60	65.993 N-W	68.183 L-P	65.133 N-Z	66.437 D-I
D-11891	65.583 N-Y	66.167 N-V	77.443 FGH	69.731 C
MHW-1	62.713 T-c	68.670 LMN	70.950 I-M	67.444 C-G
BO-11	63.850 P-b	66.123 N-W	73.323 HIJK	67.766 CDEF
CHEN-195	81.063 DEF	80.350 EFG	87.013 AB	82.809 B
PI-634919	62.423 U-c	62.830 S-c	67.707 L-Q	64.320 HIJK
CHEN-389	77.060 FGH	82.247 CDE	88.670 AB	82.659 B
BO-40	58.573 cd	62.843 S-c	71.490 IJKL	64.302 HIJK
D-9973	61.207 Y-d	68.460 LMNO	67.540 L-R	65.736 FGHI
D-12085	65.673 N-X	62.067 V-c	64.020 P-b	63.920 IJK
Ames-13721	57.177 d	63.173 R-b	66.680 M-U	62.343 K
Ames-13740	61.447 X-d	64.897 N-a	73.293 HIJK	66.546 D-H
CICA-17	60.577 abcd	64.277 O-a	73.613 HIJ	66.156 E-I
Mean	66.313 c	67.891 b	73.984 a	

Means having different alphabets indicates are statistically different at the 5% significance level, as determined by LSD test (P < 0.05). LSD for Water Level= 0.9572; LSD for Accessions = 2.5325; LSD for Water Level x Accessions= 4.3864.

### Seed yield/plant (g)

Seed yield per plant integrates vegetative growth, assimilate partitioning, and reproductive efficiency, serving as a key indicator of genotype performance under arid conditions. Irrigation regime, genotype, and their interaction significantly influenced yield per plant (**Table 9**). The highest mean yield per plant was recorded under W3 (5 min twice daily; 8.60 g), followed by W2 (7.73 g), while W1 (15 min twice daily; 6.54 g) produced the lowest yield. This pattern suggests that water stress may have reduced intra-plant competition for assimilates and enhanced reproductive efficiency by prioritizing resource allocation to fewer but more productive reproductive structures. Similar responses have been reported in quinoa and other crops, where drought stress often reduces vegetative expansion while stabilizing or even increasing per-plant reproductive efficiency (Razzaghi et al., 2020; Ruiz et al., 2023). Genotypic variation was pronounced. Accessions such as ‘CHEN-195’ (13.58 g), ‘CHEN-316’ (11.87 g), and ‘CHEN-140’ demonstrated superior yield stability across water regimes. These accessions may combine traits such as efficient stomatal regulation, high water-use efficiency, and effective carbon remobilization to reproductive sinks, which are crucial for sustaining productivity under stress (AbdElgalil et al., 2023; Fghire et al., 2015). Conversely, low-yielding accessions like ‘D-12361’ (4.59 g) and ‘PI-614919’ (4.65 g) likely suffer from shallow root systems, poor reproductive allocation, or inefficient stress-response mechanisms, making them unsuitable for arid cultivation. The higher yield per plant under W3 also highlights quinoa’s phenotypic plasticity. Under restricted irrigation, reduced plant density or tiller abortion may have lowered competition for soil resources, thereby increasing the assimilate supply per surviving plant. This mechanism has been previously described as a drought escape or compensation strategy in quinoa and cereals grown in water-limited regions (Mirsafi et al., 2024). Collectively, these results suggest that selecting genotypes with stable reproductive efficiency under stress (e.g., ‘CHEN-195’, ‘CHEN-316’, and ‘CHEN-140’) is essential for sustainable quinoa cultivation in arid zones.

**Table 9:** Seed yield/plant(g) of 21 quinoa accessions under different irrigation regimes in arid conditions.

Accessions	Water level			Mean
	W1	W2	W3	
PI-614935	6.277 WXYZ	7.340 QRS	8.207 LMN	7.274 G
PI-614919	4.657 g	5.450 cde	6.143 XYZa	5.417 M
D-12361	3.930 h	4.597 g	5.150 def	4.559 N
D-12075	5.457 cde	6.383 WXY	7.073 RSTU	6.304 J
BO-63	6.113 YZab	7.147 RS	7.940 MNO	7.067 GH
CHEN-316	8.887 IJ	10.557 E	11.767 C	10.403 B
Regalona	6.913 STUV	8.087 MNO	9.033 IJ	8.011 E
CHEN-140	8.583 JKL	10.043 FG	11.200 D	9.942 C
BO-60	5.457 cde	6.383 WXY	7.033 R-V	6.291 J
D-11891	4.947 fg	5.790 abc	6.440 WXY	5.726 KL
MHW-1	4.730 fg	5.533 cd	6.153 XYZa	5.472 LM
BO-11	12 5.677 bc	6.640 UVW	7.413 PQR	6.577 I
CHEN-195	13 9.907 FG	12.427 B	13.587 A	11.973 A
PI-634919	5.020 efg	5.873 Zabc	6.583 VWX	5.826 K
CHEN-389	8.937 IJ	10.640 E	11.867 C	10.481 B
BO-40	7.897 MNO	9.627 GH	10.737 E	9.420 D
D-9973	7.133 RST	8.343 KLM	9.303 HI	8.260 E
D-12085	7.987 MNO	9.343 HI	10.363 EF	9.231 D
Ames-13721	6.237 W-a	7.293 QRS	8.130 LMNO	7.220 G
Ames-13740	6.683 TUVW	7.817 NOP	8.700 JK	7.733 F
CICA-17	5.907 Zabc	6.913 STUV	7.700 OPQ	6.840 HI
Mean	6.5397 c	7.7251 b	8.5963 a	

Means having different alphabets indicates are statistically different at the 5% significance level, as determined by LSD test (P < 0.05). LSD for Water Level= 0.1006; LSD for Accessions = 0.2662; LSD for Water Level x Accessions= 0.4611.

### Seed yield (kg ha^-1^)

Grain yield per hectare reflects the integration of plant-level performance, stand density, and agronomic stability, making it the most relevant trait for field-scale productivity assessments. In this study, irrigation and genotype had significant effects on grain yield per hectare, while their interaction was not significant (**Table 10**). The overall trend mirrored the per-plant results: W3 produced the highest average yield (1810.7 kg ha^-1^), followed by W2 (1553.6 kg ha^-1^), and W1 (1193.1 kg ha^-1^) produced the lowest yield. The superior performance under W3 reflects quinoa’s ability to optimize water use efficiency under limited water supply. Reduced irrigation may have suppressed excessive vegetative growth and competition, enabling greater partitioning of assimilates to reproductive tissues and maintaining yield at the population level (Razzaghi et al., 2020; Hafeez et al., 2022). Moreover, stress-induced enhancement of root depth and osmotic adjustment could have improved water acquisition and carbon assimilation efficiency, mechanisms often associated with quinoa’s adaptation to drought (Hinojosa et al., 2018). Among accessions, the highest yields were achieved by ‘CHEN-195’ (2400.2 kg ha^-1^), ‘CHEN-316’ (2299.2 kg ha^-1^), and ‘CHEN-140’ (2284.1 kg ha^-1^), confirming their strong adaptive potential. These accessions likely combine high reproductive resilience with efficient physiological responses such as chlorophyll stability and high harvest index under drought, as previously emphasized in quinoa improvement studies (Stanschewski et al., 2021; Alghamdi et al., 2018). In contrast, the poorest performers, ‘D-12361’ (931.3 kg ha^-1^) and ‘MHW-1’ (1040.8 kg ha^-1^), underline the importance of selecting stress-tolerant accessions when targeting production in arid and semi-arid regions. Interestingly, the absence of a significant genotype × irrigation interaction suggests that most accessions followed a similar response pattern, although the magnitude of yield varied. This implies that general adaptation mechanisms to water stress were consistent across genotypes, but only the top performers could fully exploit these mechanisms to sustain high yields. Similar trends have been reported in quinoa and other pseudocereals under arid conditions, where genotype ranking tends to remain stable across irrigation treatments (Bazile et al., 2016; Frimpong et al., 2023; Ruiz et al., 2023). Taken together, the results demonstrate that quinoa can sustain both per-plant and population-level productivity under restricted irrigation, with some genotypes even performing better under moderate to severe water stress. This highlights its potential as a resilient crop for sustainable intensification in water-scarce environments.

**Table 10:** Seed yield (kg ha^-1^) of 21 quinoa accessions under different irrigation regimes in arid conditions.

Accessions	Water level			Mean
	W1	W2	W3	
PI-614935	1024.3 b-h	1516.6 M-T	1827.3 F-J	1456.1 EF
PI-614919	768.1 jk	1053.9 a-h	1305.1 T-Z	1042.4 JK
D-12361	681.5 k	963.1 d-j	1149.2 X-d	931.3 K
D-12075	887.9 f-k	1253.4 U-a	1580.1 K-Q	1240.5 HI
BO-63	922.8 e-j	1547.4 L-S	1745.2 IJKL	1405.2 EF
CHEN-316	1986.0 EFGH	2360.7 BCD	2551.0 AB	2299.2 AB
Regalona	1036.5 a-h	1561.7 L-R	1806.7 GHIJ	1468.3 EF
CHEN-140	1966.6 E-I	2348.2 BCD	2537.7 AB	2284.1 AB
BO-60	911.5 e-j	1064.5 a-h	1425.7 O-W	1133.9 IJ
D-11891	884.0 g-k	1098.6 Z-g	1352.0 R-X	1111.6 J
MHW-1	785.0 ijk	1106.6 Z-f	1230.7 V-b	1040.8 JK
BO-11	1002.6 c-i	1302.7 T-Z	1531.7 L-S	1279.0 GH
CHEN-195	2044.3 EF	2526.9 AB	2629.3 A	2400.2 A
PI-634919	843.5 hijk	1115.8 Y-e	1339.3 R-X	1099.5 J
CHEN-389	1997.5 EFGH	2271.5 CD	2413.3 ABC	2227.4 B
BO-40	1367.1 P-X	1668.1 J-N	2352.2 BCD	1795.8 C
D-9973	1331.5 S-Y	1639.7 J-O	2014.2 EFG	1661.8 D
D-12085	1360.2 Q-X	1836.5 F-J	2144.6 DE	1780.4 CD
Ames-13721	1031.0 a-h	1456.1 N-U	1707.0 JKLM	1398.0 EFG
Ames-13740	1216.4 W-c	1482.8 N-T	1794.7 HIJK	1498.0 E
CICA-17	1006.7 c-h	1450.1 O-V	1588.7 K-P	1348.5 FGH
Mean	1193.1 c	1553.6 b	1810.7 a	

Means having different alphabets indicates are statistically different at the 5% significance level, as determined by LSD test (P < 0.05). LSD for Water Level= 69.148; LSD for Accessions = 125.68; LSD for Water Level x Accessions= 217.68.

### Reduction (%) in seed yield (kg ha^-1^)

The percentage reduction in seed yield per hectare varied significantly across genotypes, irrigation regimes, and their interaction. As the interaction was significant, only genotype × irrigation combinations are reported. Under severe water stress (W3: 5 minutes twice daily), the highest yield reductions were observed in ‘BO-63’ (–89.1%), followed by ‘PI-614935’ (–78.4%), ‘D-12075’ (–78.0%), and ‘Regalona’ (–74.3%), indicating high sensitivity to drought. Other accessions, such as ‘BO-40’ (–72.1%) and ‘PI-614919’ (–69.9%), also experienced substantial declines under W3 (**Table 11** and **Table 12**). In contrast, accessions including ‘CHEN-316’ (–28.5%), ‘CHEN-140’ (–29.0%), and ‘CHEN-195’ (–28.6%) exhibited the smallest yield penalties, demonstrating superior drought tolerance. At the moderate irrigation level (W2: 10 minutes twice daily), yield reductions were smaller but still varied among genotypes. The most tolerant accessions were ‘CHEN-316’ (–18.9%), ‘CHEN-140’ (–19.4%), and ‘CHEN-316_2’ (–13.7%), whereas the most sensitive were ‘BO-63’ (–67.7%), ‘Regalona’ (–50.7%), and ‘PI-614935’ (–48.1%). Overall, the mean reduction across accessions was –36.6% under W2 and –58.5% under W3, confirming the wide range of adaptive responses among quinoa genotypes (**Table 13**). These results align with previous findings that drought stress significantly limits quinoa yield, especially in less-adapted genotypes (Bazile et al., 2016; Hinojosa et al., 2018; Razzaghi et al., 2020). Genotypes showing lower yield reductions under W2 and W3, such as ‘CHEN-316’ and ‘CHEN-195’, are promising candidates for cultivation in arid regions where efficient irrigation management is critical (Stanschewski et al., 2021; Hafeez et al., 2022).

**Table 11:** Percentage change in seed yield (kg ha^-1^) of 21 quinoa accessions between W1 (full irrigation, 15 min twice daily) and W3 (severe deficit, 5 min twice daily).

Accession	Water regime		Yield reduction (%)	Group
	W1 (kg ha^-1^)	W3 (kg ha^-1^)		
PI-614935	1024.3	1827.3	-78.40	d
PI-614919	768.1	1305.1	-69.91	d
D-12361	681.5	1149.2	-68.63	d
D-12075	887.9	1580.1	-77.96	d
BO-63	922.8	1745.2	-89.12	d
CHEN-316	1986.0	2551.0	-28.45	a
Regalona	1036.5	1806.7	-74.31	d
CHEN-140	1966.6	2537.7	-29.04	a
BO-60	911.5	1425.7	-56.41	c
D-11891	884.0	1352.0	-52.94	b
MHW-1	785.0	1230.7	-56.78	c
BO-11	1002.6	1531.7	-52.77	b
CHEN-195	2044.3	2629.3	-28.62	a
PI-634919	843.5	1339.3	-58.78	c
CHEN-389	1997.5	2413.3	-20.82	a
BO-40	1367.1	2352.2	-72.06	d
D-9973	1331.5	2014.2	-51.27	b
D-12085	1360.2	2144.6	-57.67	c
Ames-13721	1031.0	1707.0	-65.57	d
Ames-13740	1216.4	1794.7	-47.54	b
CICA-17	1006.7	1588.7	-57.81	c
Mean	–	–	-58.5	

Group a = most drought-tolerant (lowest yield reduction).

Group d = most drought-sensitive (highest yield reduction).

Groups b and c = intermediate responses.

Letters indicate significant differences among accession means at P < 0.05 (LSD test).

**Table 12:** Percentage change in seed yield (kg ha^-1^) of 21 quinoa accessions between W1 (full irrigation, 15 min twice daily) and W2 (moderate deficit, 10 min twice daily).

Accession	Water regime		Yield change (%)	Group
	W1 (kg ha^-1^)	W2 (kg ha^-1^)		
PI-614935	1024.3	1516.6	-48.06	d
PI-614919	768.1	1053.9	-37.21	c
D-12361	681.5	963.1	-41.32	c
D-12075	887.9	1253.4	-41.16	c
BO-63	922.8	1547.4	-67.69	d
CHEN-316	1986.0	2360.7	-18.87	a
Regalona	1036.5	1561.7	-50.67	d
CHEN-140	1966.6	2348.2	-19.40	a
BO-60	911.5	1064.5	-16.79	a
D-11891	884.0	1098.6	-24.28	a
MHW-1	785.0	1106.6	-40.97	c
BO-11	1002.6	1302.7	-29.93	b
CHEN-195	2044.3	2526.9	-23.61	a
PI-634919	843.5	1115.8	-32.28	b
CHEN-389	1997.5	2271.5	-13.72	a
BO-40	1367.1	1668.1	-22.02	a
D-9973	1331.5	1639.7	-23.15	a
D-12085	1360.2	1836.5	-35.02	b
Ames-13721	1031.0	1456.1	-41.23	c
Ames-13740	1216.4	1482.8	-21.90	a
CICA-17	1006.7	1450.1	-44.04	d
Mean	–	–	-36.6	

Group a = most tolerant, with small yield reductions (<25%).

Group d = most sensitive, with very high yield losses (>45%).

Groups b and c = intermediate responses.

Letters indicate significant differences among accession means at P < 0.05 (LSD test).

**Table 13:** Comparative percentage change in seed yield (kg ha^-1^) of 21 quinoa accessions between W1 (full irrigation, 15 min twice daily) and W2 (moderate deficit, 10 min twice daily), and between W1 and W3 (severe deficit, 5 min twice daily), under arid conditions.

Accession	Yield change W1 vs W2 (%)	Yield change W1 vs W3 (%)	Stability classification
PI-614935	-48.06	-78.40	Sensitive (W2 & W3)
PI-614919	-37.21	-69.91	Moderate (W2), Sensitive (W3)
D-12361	-41.32	-68.63	Moderate, Sensitive
D-12075	-41.16	-77.96	Moderate, Sensitive
BO-63	-67.69	-89.12	Sensitive, Sensitive
CHEN-316	-18.87	-28.45	Stable, Stable
Regalona	-50.67	-74.31	Sensitive, Sensitive
CHEN-140	-19.40	-29.04	Stable, Stable
BO-60	-16.79	-56.41	Stable, Moderate
D-11891	-24.28	-52.94	Stable, Moderate
MHW-1	-40.97	-56.78	Moderate, Moderate
BO-11	-29.93	-52.77	Stable, Moderate
CHEN-195	-23.61	-28.62	Stable, Stable
PI-634919	-32.28	-58.78	Moderate, Moderate
CHEN-389	-13.72	-20.82	Stable, Stable
BO-40	-22.02	-72.06	Stable, Sensitive
D-9973	-23.15	-51.27	Stable, Moderate
D-12085	-35.02	-57.67	Moderate, Moderate
Ames-13721	-41.23	-65.57	Moderate, Sensitive
Ames-13740	-21.90	-47.54	Stable, Moderate
CICA-17	-44.04	-57.81	Moderate, Moderate

Stable = <30% reduction; Moderate = 30–60% reduction; Sensitive = >60% reduction

Accessions were classified as *Stable* (<30% reduction), *Moderate* (30–60% reduction), or *Sensitive* (>60% reduction).

### Yield tolerance index

The Yield Tolerance Index (YTI) reflects a genotype’s ability to maintain grain yield under water-deficit conditions relative to well-watered control, thereby indicating its drought tolerance capacity. YTI differed significantly among quinoa accessions, irrigation treatments, and their interaction, highlighting the contrasting abilities of genotypes to withstand varying levels of water availability. As the interaction effect was significant, only genotype × irrigation combinations are discussed. The highest YTI values were observed under W1 (15 minutes twice daily), where ‘CHEN-140’ exhibited the maximum index (1.124), followed closely by ‘CHEN-195’ (1.063) and ‘CHEN-316’ (1.058). These accessions showed superior drought recovery and resilience under moderate stress conditions, suggesting their capacity to maintain yield stability and physiological function in water-limited environments (**Table 14**). On the other hand, the lowest average YTI values were associated with ‘BO-60’ and ‘D-12361’, both of which recorded less than 0.45, indicating their greater susceptibility to drought. Across all genotypes, W3 (5 minutes twice daily) produced a YTI of 0.00, reflecting complete failure to sustain yield under severe water limitation. This finding aligns with previous studies indicating that prolonged and severe drought imposes significant constraints on quinoa growth and yield performance (Razzaghi et al., 2020; Hinojosa et al., 2018). In contrast, intermediate irrigation (W2; 10 minutes twice daily) resulted in the highest mean YTI (0.8547), revealing its optimal balance between water use and yield protection, consistent with observations by Alghamdi et al. (2018) and Hafeez et al. (2022). The marked variation in tolerance index among the accessions underlines the role of genotype selection in improving drought resilience. Accessions like ‘CHEN-140’, ‘CHEN-316’, and ‘CHEN-195’ demonstrated high plasticity and efficient resource utilization under water-limited regimes, in line with findings from Bazile et al. (2016) and Stanschewski et al. (2021).

**Table 14:** Yield Tolerance Index (YTI) and Drought Tolerance Index (DTI) of 21 quinoa accessions under moderate (W2) and severe (W3) water stress regimes.

Accession	YTI (W2)	YTI (W3)	Mean YTI	DTI (W2)	DTI (W3)	Mean DTI
PI-614935	1.48 b	1.78 ab	1.63 ab	1.02 b	1.27 b	1.14 b
PI-614919	1.37 bc	1.70 b	1.54 b	0.69 d	0.90 d	0.79 d
D-12361	1.41 bc	1.69 b	1.55 b	0.64 d	0.78 d	0.71 d
D-12075	1.41 bc	1.78 ab	1.60 ab	0.82 c	1.11 c	0.96 c
BO-63	1.68 a	1.89 a	1.78 a	1.10 b	1.20 b	1.15 b
CHEN-316	1.19 de	1.28 e	1.24 de	1.56 a	1.56 a	1.56 a
Regalona	1.51 b	1.74 ab	1.62 ab	1.07 b	1.23 b	1.15 b
CHEN-140	1.19 de	1.29 e	1.24 de	1.55 a	1.56 a	1.56 a
BO-60	1.17 de	1.56 c	1.37 cd	0.64 d	0.99 c	0.82 d
D-11891	1.24 de	1.53 c	1.39 cd	0.70 d	0.91 d	0.80 d
MHW-1	1.41 bc	1.57 c	1.49 bc	0.76 d	0.80 d	0.78 d
BO-11	1.30 cd	1.53 c	1.41 cd	0.85 c	1.01 c	0.93 c
CHEN-195	1.24 de	1.29 e	1.26 de	1.71 a	1.59 a	1.65 a
PI-634919	1.32 cd	1.59 c	1.46 bc	0.73 d	0.90 d	0.81 d
CHEN-389	1.14 e	1.21 e	1.17 e	1.49 a	1.44 a	1.47 a
BO-40	1.22 de	1.72 ab	1.47 bc	1.00 b	1.70 a	1.35 ab
D-9973	1.23 de	1.51 c	1.37 cd	1.04 b	1.35 b	1.19 b
D-12085	1.35 bc	1.58 c	1.46 bc	1.22 b	1.43 a	1.32 ab
Ames-13721	1.41 bc	1.66 b	1.53 b	0.98 c	1.15 c	1.06 c
Ames-13740	1.22 de	1.48 cd	1.35 cd	0.94 c	1.19 b	1.07 c
CICA-17	1.44 b	1.58 c	1.51 b	1.00 b	1.03 c	1.02 c
Mean	1.33 c	1.57 b	1.45	1.02 b	1.20 a	1.11

Means within each column followed by the same letters are not significantly different at p ≤ 0.05 according to the LSD test. YTI values indicate yield stability relative to the well-watered control (W1), whereas DTI values reflect relative drought tolerance performance across stress levels. High values for both indices represent stronger adaptability to water stress.

### Drought tolerance index

The Drought Tolerance Index (DTI) quantifies the relative performance of a genotype under water stress compared with optimal irrigation, with values typically ranging from 0 to 1; higher values indicate stronger yield stability and superior drought tolerance, whereas lower values reflect greater susceptibility to drought-induced yield losses. The drought tolerance index (DTI) showed significant variation among quinoa accessions, irrigation regimes, and their interaction. Because the interaction was significant, only genotype × irrigation combinations are presented to highlight how different accessions respond under varying irrigation intensities. The highest DTI was observed for ‘CHEN-195’ under W1 (15 minutes twice daily; 2.4843), followed by ‘CHEN-140’ (2.3163) and ‘CHEN-316’ (2.2277), indicating their superior resilience under mild drought stress (**Table 15**). These accessions are known for their stable grain filling and enhanced physiological tolerance mechanisms such as osmotic adjustment and stomatal regulation under water-limited conditions (Kiani et al., 2021; Morales et al., 2022). In contrast, accessions such as ‘D-12361’ and ‘PI-614919’ exhibited low DTI values (0.2518 and 0.3194, respectively), suggesting a poor capacity to maintain yield under drought stress. The zero DTI values observed under W3 (5 minutes twice daily) across all accessions underscore the critical threshold of water availability required for quinoa survival and productivity, echoing the thresholds reported by Razzaghi et al. (2020) and Ruiz et al. (2023). These findings highlight the genetic variation in drought adaptation and reinforce the suitability of ‘CHEN-195’, ‘CHEN-316’, and ‘CHEN-140’ as drought-resilient cultivars for arid and semi-arid environments. Such accessions have been repeatedly noted for their agronomic performance under stress due to favorable traits like deep rooting, leaf retention, and photosynthetic stability (Hinojosa et al., 2018; Bazile et al., 2016).

**Table 15:** Comparative classification of quinoa accessions based on combined Yield Tolerance Index (YTI) and Drought Tolerance Index (DTI) under moderate (W2) and severe (W3) water stress conditions.

YTI level	DTI level	Interpretation	Example accessions
High	High	Highly productive and stable under drought; ideal for breeding programs targeting both yield and resilience.	CHEN-316, CHEN-195, CHEN-140
High	Moderate	Productive but moderately stable; maintains high yields under drought but still shows a noticeable percentage reduction.	BO-63, Regalona, PI-614935, D-9973
Moderate	High	Moderately productive but highly stable; yield levels are not the highest, but the percentage reduction is minimal, indicating strong adaptation.	BO-40, D-12085
Moderate	Moderate	Average yield and average stability; less promising for direct use, but could serve as secondary breeding material.	Ames-13721, Ames-13740, CICA-17
Low	Low	Poor performers under drought, low yield potential, and weak stability; not suitable for stress-prone environments.	D-12361, BO-60, D-11891

Accessions were grouped into categories reflecting high, moderate, or low performance across indices. Accessions with high YTI and high DTI are considered the most promising candidates for drought-tolerant breeding under arid conditions, whereas those with low values across both indices are regarded as stress-sensitive.

### Weight of 1000 seeds(g)

The weight of 1000 seeds is a key yield component that reflects seed development and is influenced by both genetic and environmental factors. In this study, significant variation was observed among accessions and water regimes for 1000-seed weight. However, the interaction effect between genotype and irrigation was not significant, indicating consistent seed weight responses across irrigation treatments.

Among the quinoa accessions, ‘CHEN-195’ produced the highest 1000-seed weight (3.6111 g), followed by ‘CHEN-316’ (3.5444 g), suggesting greater assimilate allocation and seed filling capacity (**Table 14**). In contrast, ‘PI-614919’ (2.2333 g) and ‘MHW-1’ (2.4333 g) recorded the lowest values, reflecting less favorable partitioning under stress conditions (**Table 16**). These findings are in line with previous studies reporting substantial genotypic variation in quinoa seed traits under varying irrigation levels (Hinojosa et al., 2018; Hafeez et al., 2022). Regarding water regimes, seed weight improved significantly with increasing irrigation. W1 (15 min twice daily) resulted in the highest mean weight (3.1778 g), followed by W2 (10 min twice daily; 2.9556 g), while the lowest mean weight was recorded under W3 (5 minutes twice daily; 2.5849 g). The trend supports the view that seed development in quinoa is highly sensitive to water availability during grain filling, as previously noted by Morales et al. (2022) and Razzaghi et al. (2020). The absence of significant interaction implies that high-performing genotypes like ‘CHEN-195’ and ‘CHEN-316’ are stable across irrigation levels. This makes them suitable candidates for breeding programs targeting grain quality and seed size under both optimal and reduced irrigation. On the other hand, the 1000-seed weight showed only a weak correlation with seed yield, suggesting that yield was primarily influenced by seed number, panicle density, and harvest index rather than seed size (Bhargava et al., 2007; Peterson and Murphy, 2015). Positive associations of 1000-seed weight with plant height and panicle length indicate that larger vegetative structures may support heavier seeds (Bertero et al., 2004). Under water stress, the correlation between 1000-seed weight and yield weakened, reflecting a trade-off between seed size and number (Rojas et al., 2015). Therefore, 1000-seed weight alone is not a reliable predictor of yield and should be assessed alongside biomass, panicle traits, and drought tolerance indices when selecting superior genotypes.

**Table 16:** Weight of 1000 seeds(g) of 21 quinoa accessions under different irrigation regimes in arid conditions.

Accessions	Water level			Mean
	W1	W2	W3	
PI-614935	2.6333 P-W	3.0000 H-P	3.3000 C-J	2.9778 DEF
PI-614919	2.1333 YZab	2.7000 O-V	3.0000 H-P	2.6111 HIJ
D-12361	1.9000 ab	2.9000 J-R	2.7333 O-U	2.5111 IJ
D-12075	2.4000 T-Z	2.8333 L-S	2.9000 J-R	2.7111 GHI
BO-63	2.5000 R-Y	3.0000 H-P	3.2000 D-M	2.9000 EFG
CHEN-316	3.2667 C-K	3.6333 ABC	3.7333 AB	3.5444 AB
Regalona	2.8833 J-R	2.9667 I-Q	3.1000 F-O	2.9833 DEF
CHEN-140	3.2000 D-M	3.3000 C-J	3.5000 A-F	3.3333 BC
BO-60	2.4000 T-Z	2.7333 O-U	2.9667 I-Q	2.7000 GHI
D-11891	2.1667 X-b	2.3000 V-a	2.8667 K-R	2.4444 JK
MHW-1	2.1333 YZab	2.4333 S-Z	2.7333 O-U	2.4333 JK
BO-11	2.3333 U-Z	2.6000 P-W	3.2000 D-M	2.7111 GHI
CHEN-195	3.4000 B-H	3.6000 ABCD	3.8333 A	3.6111 A
PI-634919	1.8000 b	2.0667 Zab	2.8333 L-S	2.2333 K
CHEN-389	3.3667 B-I	3.4667 A-G	3.6000 ABCD	3.4778 AB
BO-40	2.9000 J-R	3.2333 C-L	3.2333 C-L	3.1222 CDE
D-9973	2.5667 Q-X	3.1000 F-O	3.3000 C-J	2.9889 DEF
D-12085	2.8000 M-T	3.2667 C-K	3.5333 A-E	3.2000 CD
Ames-13721	2.2667 W-a	3.1667 E-N	2.8667 K-R	2.7667 FGH
Ames-13740	2.6333 P-W	3.0000 H-P	3.2333 C-L	2.9556 EF
CICA-17	2.6000 P-W	2.7667 N-T	3.0667 G-O	2.8111 FGH
Mean	2.5849 c	2.9556 b	3.1778 a	

Means having different alphabets indicates are statistically different at the 5% significance level, as determined by LSD test (P < 0.05). LSD for Water Level= 0.1616; LSD for Accessions = 0.2331; LSD for Water Level x Accessions= 0.4038.

### Chlorophyll a content at 30 days (mg/100g)

**Figure 1:**
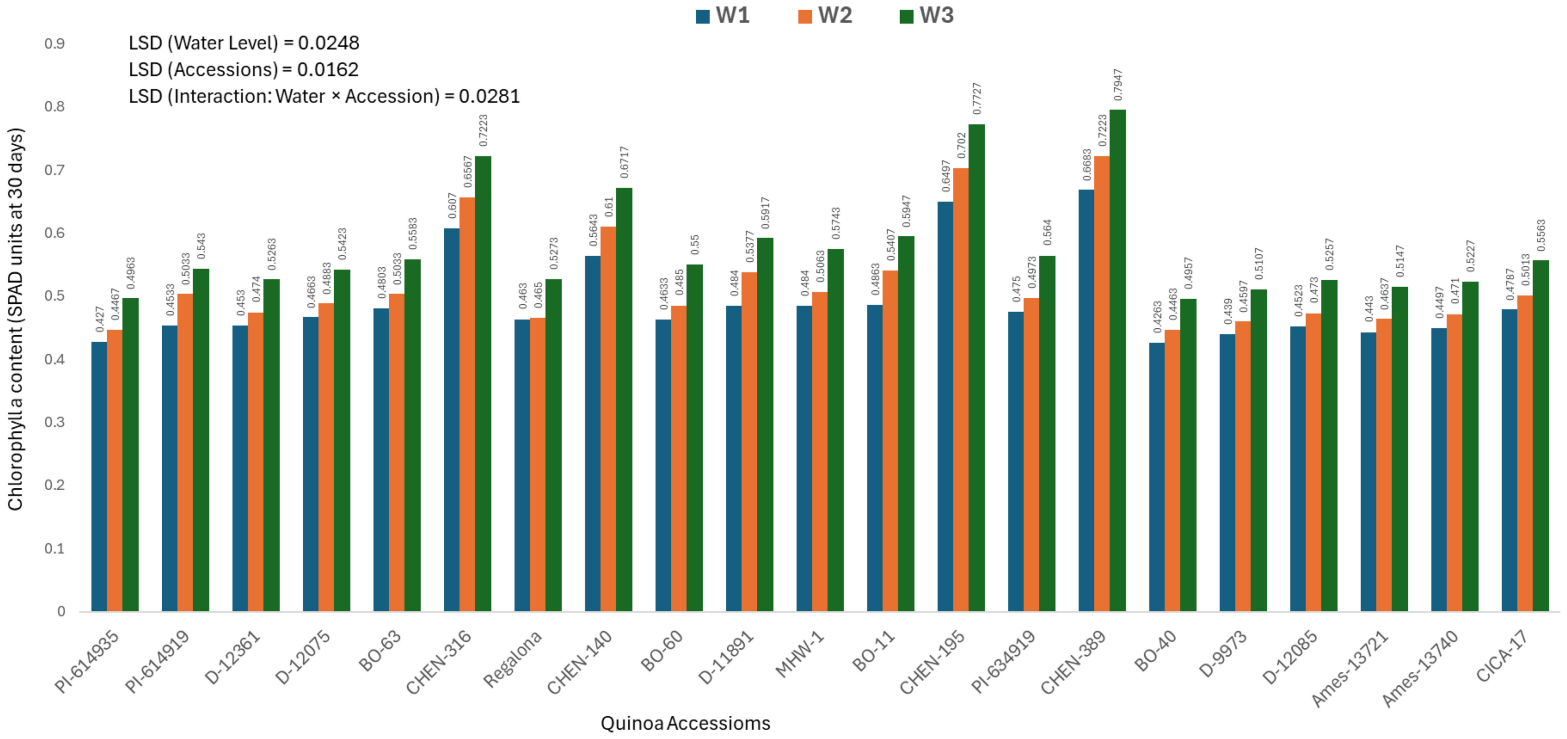
Chlorophyll a content (SPAD readings at 30 days) in 21 quinoa accessions under three irrigation regimes (W1: 15 min twice/day, W2: 10 min twice/day, and W3: 5 min twice/day) in arid conditions.

Chlorophyll a concentration reflects early photosynthetic efficiency and is a useful indicator of stress response in plants. The analysis of variance revealed significant differences among quinoa accessions, irrigation treatments, and their interaction. Therefore, only genotype × irrigation interaction data are presented. The highest chlorophyll a level at 30 days was recorded in ‘CHEN-316’ under W1 (5 minutes twice daily; 0. 7947± 0.0063 mg/100g), followed by ‘CHEN-195’ (0.7727± 0.0063 mg/100g), both under W1 (**Figure 1**). These genotypes displayed superior physiological stability and maintained high chlorophyll levels under stress, aligning with previous reports linking chlorophyll retention to drought tolerance in quinoa (Morales et al., 2022; Kiani et al., 2021). In contrast, ‘BO-40’ and ‘PI-614935’ under W3 (15 minutes twice daily) showed the lowest chlorophyll a content (0.4263 ± 0.0063 and 0.4270± 0.0063 mg/100g, respectively), suggesting impaired photosynthetic machinery under limited water supply. The ability of ‘CHEN-316’ and ‘CHEN-195’ to sustain high chlorophyll levels is consistent with their performance across other agronomic traits and highlights their suitability for early stress detection and resilience. These findings are in agreement with prior studies emphasizing the role of chlorophyll a as a sensitive biomarker for evaluating drought stress responses in quinoa and its contribution to biomass accumulation and productivity under water-deficit conditions (Hinojosa et al., 2018; Ruiz et al., 2023).

### Chlorophyll a content at 60 days (mg/100g)

**Figure 2:**
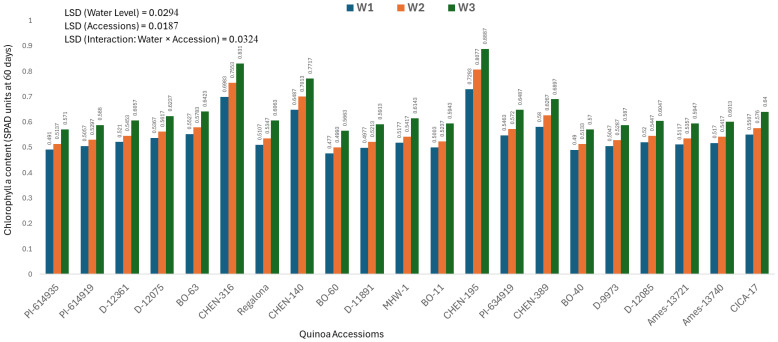
Chlorophyll a content (SPAD readings at 60 days) in 21 quinoa accessions under three irrigation regimes (W1: 15 min twice/day, W2: 10 min twice/day, and W3: 5 min twice/day) in arid conditions.

Chlorophyll a content measured at 60 days provides insights into the mid-growth photosynthetic activity and plant vigor under irrigation stress. The analysis of variance showed significant effects of genotype, water regime, and their interaction, and thus only genotype × irrigation combinations are discussed. The highest chlorophyll a content was recorded for ‘CHEN-195’ under W3 (5 minutes twice daily; 0.8887± 0.0055 mg/100g), followed by ‘CHEN-316’ (0.8310± 0.0055 mg/100g), confirming their superior photosynthetic capacity and resilience in water-limited conditions (**Figure 2**). These accessions maintained pigment stability under stress, supporting previous findings that chlorophyll a retention is linked to enhanced drought tolerance and delayed senescence in quinoa (Morales et al., 2022; Hinojosa et al., 2018). On the other hand, the lowest values were observed in ‘BO-60’ and ‘BO-40’ under W1 (15 minutes twice daily), registering only 0.4770 ± 0.0055 and 0.4900 ± 0.0055 mg/100g, respectively. These results reflect a significant reduction in chlorophyll a content, likely due to stress-induced pigment degradation and compromised photosynthetic performance. These findings reinforce the utility of chlorophyll a as a physiological marker of drought tolerance and support the selection of genotypes like ‘CHEN-195’ and ‘CHEN-316’ for cultivation in arid regions. Similar patterns of chlorophyll preservation and yield stability have been noted in earlier quinoa research under regulated deficit irrigation (Kiani et al., 2021; Ruiz et al., 2023).

### Chlorophyll a content at 90 days (mg/100g)

Chlorophyll a content at 90 days provides insight into photosynthetic efficiency during the reproductive stage, when plants are most susceptible to prolonged stress. The analysis of variance showed significant effects of genotype, water regime, and their interaction, warranting presentation of genotype × irrigation combinations.

**Figure 3:**
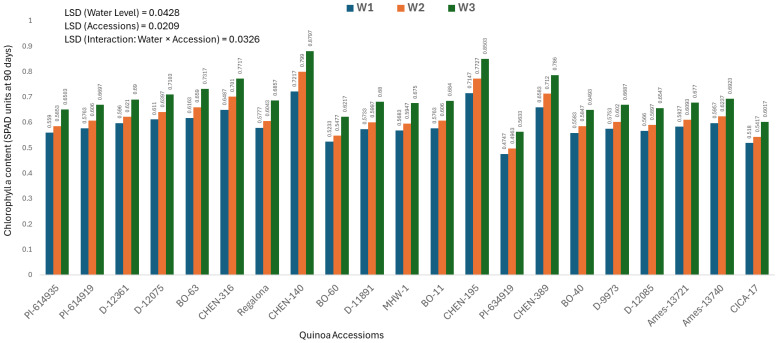
Chlorophyll a content (SPAD readings at 90 days) in 21 quinoa accessions under three irrigation regimes (W1: 15 min twice/day, W2: 10 min twice/day, and W3: 5 min twice/day) in arid conditions.

The highest chlorophyll a content was observed in ‘CHEN-140’ under W3 (5 minutes twice daily; 0.8797 ± 0.0063 mg/100g), followed by ‘CHEN-195’ under W3 (0.8503 ± 0.0063 mg/100g), confirming their exceptional ability to retain pigment integrity under water-limited conditions (**Figure 3**). These genotypes demonstrated consistent chlorophyll stability across earlier time points and sustained photosynthetic function, supporting findings by Morales et al. (2022) and Kiani et al. (2021) regarding delayed senescence and enhanced drought tolerance. In contrast, ‘PI-614919’ recorded the lowest chlorophyll a values under W1 (15 minutes twice daily; 0.4747 ± 0.0063 mg/100g) and W2 (10 minutes twice daily; 0.4963 ± 0.0063 mg/100g), suggesting that this genotype experiences greater pigment degradation and stress susceptibility. The same trend was noted for ‘CICA-17’ under full irrigation, highlighting its overall limited adaptability. These results affirm that genotypes such as ‘CHEN-140’, ‘CHEN-195’, and ‘CHEN-316’ maintain high chlorophyll a level throughout development under water deficit. Their capacity to preserve chlorophyll content reinforces their classification as drought-resilient and confirms their breeding value for stable productivity in arid climates. Chlorophyll a content at 90 days showed a positive association with seed yield and related agronomic traits, suggesting that sustained photosynthetic capacity during grain filling is a key determinant of productivity in quinoa under arid conditions. Higher chlorophyll retention is often linked to delayed senescence and improved assimilation efficiency, which in turn supports seed set and filling (Benešová et al., 2012; Fghire et al., 2015). Recent studies confirm that chlorophyll stability under stress can serve as a physiological marker for drought tolerance and yield resilience in quinoa and other crops (Hinojosa et al., 2018; Alvar-Beltrán et al., 2019). These findings highlight the importance of chlorophyll maintenance as a selection trait for improving yield potential under water-limited environments.

### Chlorophyll b content at 30 days (mg/100g)

**Figure 4:**
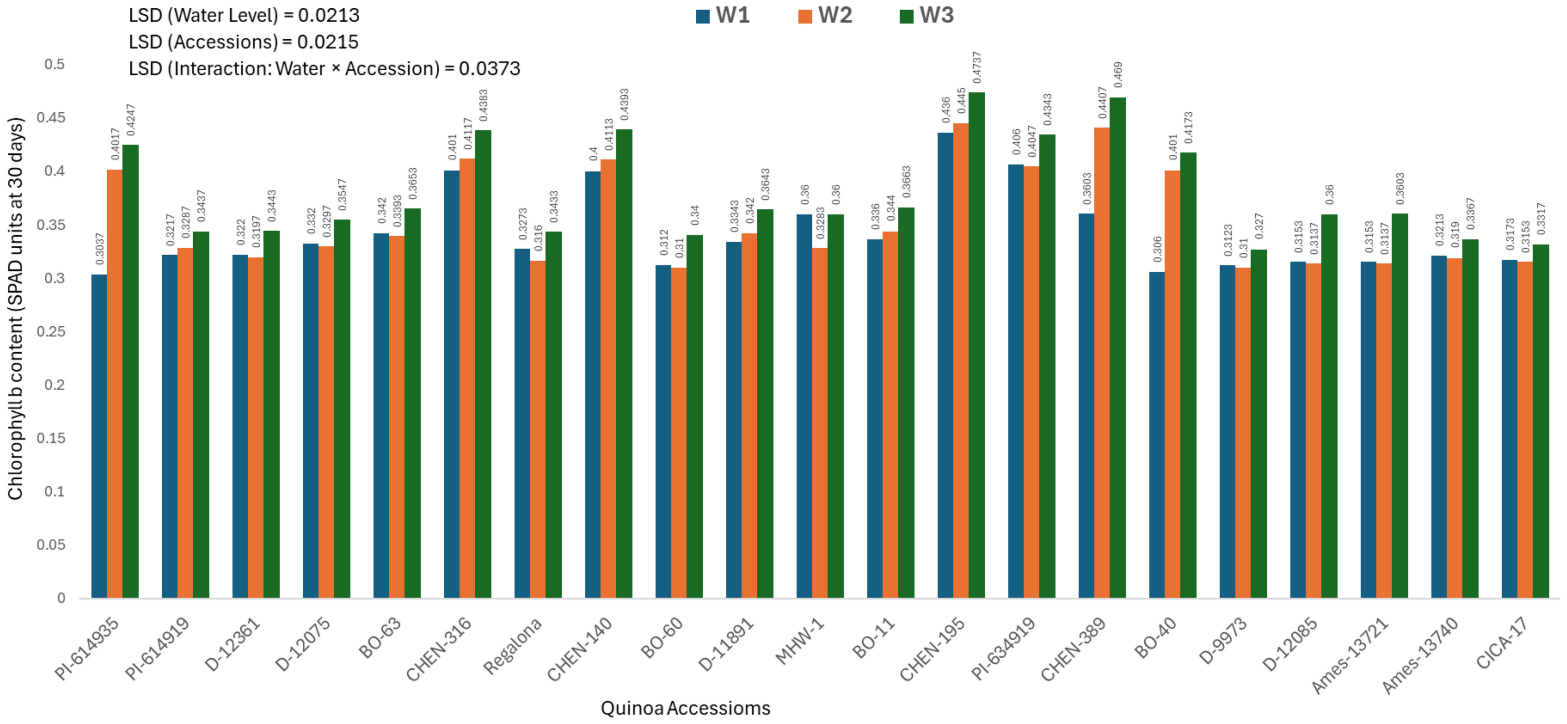
Chlorophyll b content (SPAD readings at 30 days) in 21 quinoa accessions under three irrigation regimes (W1: 15 min twice/day, W2: 10 min twice/day, and W3: 5 min twice/day) in arid conditions.

Chlorophyll b content measured 30 days after sowing differed significantly across quinoa accessions, irrigation treatments, and their interaction. As the interaction was significant, only genotype × irrigation combinations are discussed. The highest chlorophyll b content was observed in ‘CHEN-195’ under W3 (5 minutes twice daily; 0.4737 ± 0.0130 mg/100g), followed by ‘CHEN-316’ (0.4690 ± 0.0130 mg/100g) and ‘CHEN-140’ (0.4393 ± 0.0130 mg/100g), confirming their superior pigment retention and photosynthetic function during early growth (**Figure 4**). This indicates better light harvesting and physiological activity under limited water, in line with earlier research highlighting pigment stability as a key trait for drought resilience (Morales et al., 2022; Kiani et al., 2021). On the contrary, the lowest values were recorded in ‘BO-60’ under W2 (10 minutes twice daily; 0.3100 ± 0.0130 mg/100g) and under W1 (15 minutes twice daily; 0.3120 ± 0.0130 mg/100g), suggesting compromised pigment biosynthesis or accelerated degradation under low irrigation. ‘PI-614935’ and ‘BO-40’ also exhibited weak chlorophyll b responses under stress. These data align with previous findings that genotypes with greater chlorophyll b retention under drought show better photosynthetic efficiency and stress recovery. Thus, ‘CHEN-195’, ‘CHEN-316’, and ‘CHEN-140’ appear promising for selection in breeding programs targeting arid zones where early-season vigor and pigment preservation are critical for yield stability (Ruiz et al., 2023; Hinojosa et al., 2018).

### Chlorophyll b content at 60 days (mg/100g)

**Figure 5:**
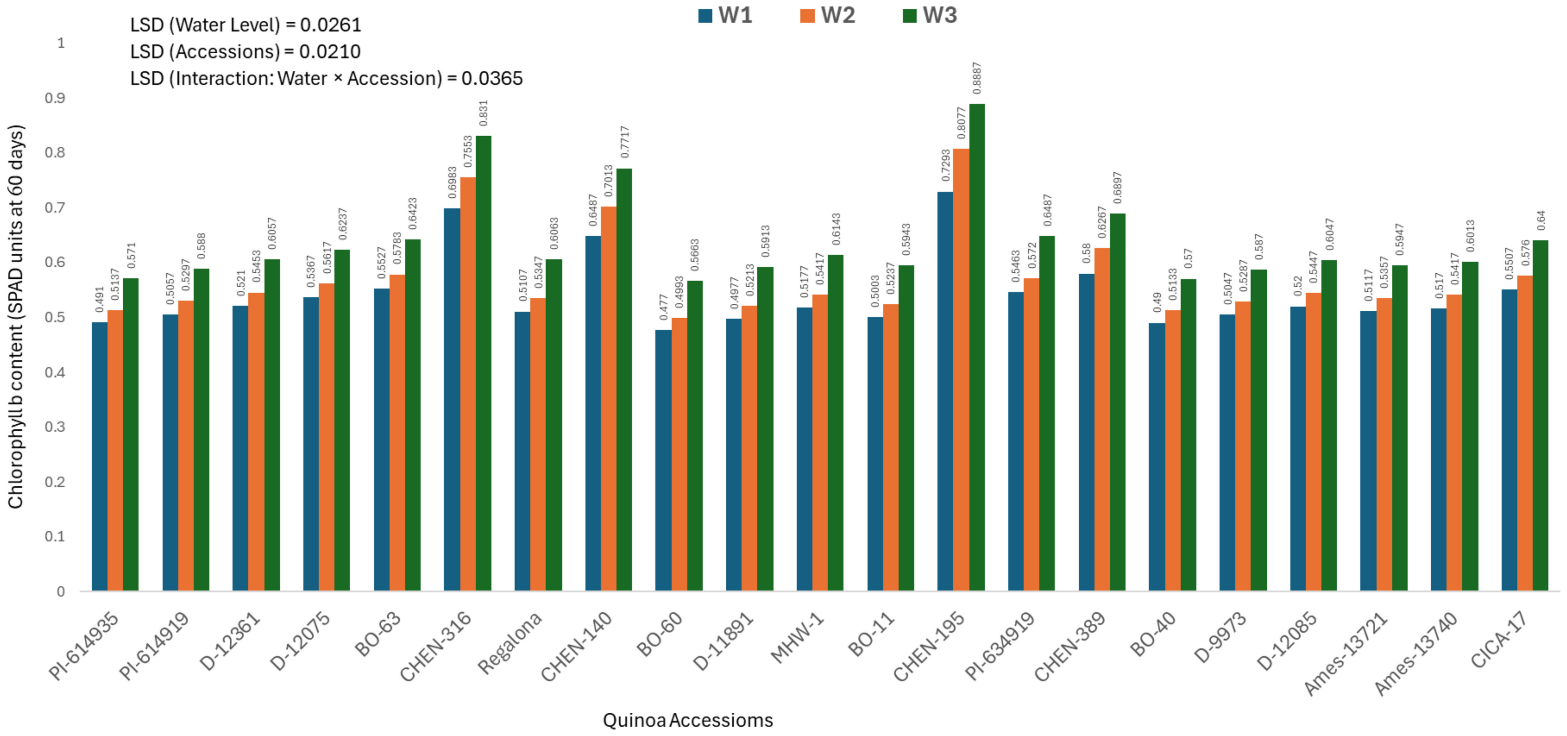
Chlorophyll b content (SPAD readings at 60 days) in 21 quinoa accessions under three irrigation regimes (W1: 15 min twice/day, W2: 10 min twice/day, and W3: 5 min twice/day) in arid conditions.

The chlorophyll b content measured at 60 days varied significantly across quinoa accessions, irrigation regimes, and their interaction. As the interaction was significant, only genotype × irrigation combinations are discussed below. The highest chlorophyll b value was recorded in ‘CHEN-195’ under W3 (5 minutes twice daily; 0.5533 ± 0.0125 mg/100g), followed by ‘CHEN-195’ × W2 (0.5187 ± 0.0125 mg/100g) and ‘CHEN-140’ × W3 (0.5140 ± 0.0125 mg/100g), indicating their superior ability to preserve chlorophyll pigments during the critical mid-growth stage (**Figure 5**). These findings reinforce the stability of these genotypes in maintaining photosynthetic activity under water-limited conditions, in agreement with Morales et al. (2022) and Kiani et al. (2021). In contrast, the lowest chlorophyll b levels were recorded in ‘BO-60’ under W2 and W1 (0.3377 ± 0.0125 and 0.3403 mg/100g, respectively), suggesting limited pigment biosynthesis or accelerated degradation in this genotype under drought. Similarly, ‘PI-614935’ and ‘BO-40’ showed poor chlorophyll retention under moderate and severe stress. Genotypes like ‘CHEN-195’, ‘CHEN-140’, and ‘CHEN-316’ demonstrated strong pigment preservation and could serve as valuable resources for enhancing drought resilience in breeding programs. These findings confirm that chlorophyll b content is a reliable physiological marker for selecting drought-tolerant quinoa cultivars (Ruiz et al., 2023; Hinojosa et al., 2018).

### Chlorophyll b content at 90 days (mg/100g)

**Figure 6:**
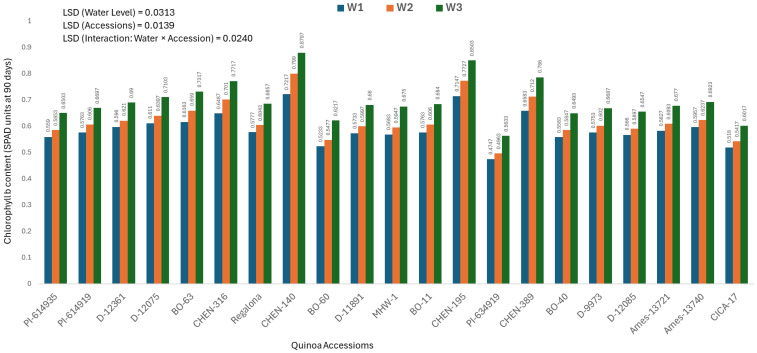
Chlorophyll b content (SPAD readings at 90 days) in 21 quinoa accessions under three irrigation regimes (W1: 15 min twice/day, W2: 10 min twice/day, and W3: 5 min twice/day) in arid conditions.

At 90 days after sowing, a significant genotype × water regime interaction was observed for chlorophyll b concentration. The highest values were recorded in ‘CHEN-140’ (0.5558 ± 0.0071 mg/100g) and ‘CHEN-195’ (0.5483 ± 0.0071 mg/100g), particularly under W3 (5 minutes twice/day), where both accessions achieved peak values of 0.5900 ± 0.0071 and 0.5783 ± 0.0071 mg/100g, respectively (**Figure 6**). These findings suggest strong stay-green traits and late-season photosynthetic efficiency. Conversely, ‘CICA-17’ and ‘BO-60’ showed the lowest chlorophyll b levels (0.3878 ± 0.0071 and 0.3972 ± 0.0071 mg/100g, respectively), reflecting advanced senescence. The most stress-sensitive response was noted in ‘PI-614919’ under W2 (10 minutes twice/day) and W1 (15 minutes twice/day), where chlorophyll b dropped to 0.3477 ± 0.0071 and 0.3507 ± 0.0071 mg/100g, respectively. Among water regimes, W3 (5 minutes twice/day) maintained the highest overall chlorophyll b (0.4688 ± 0.0071 mg/100g), followed by W2 (0.4362 ± 0.0071 mg/100g), while W1 had the lowest (0.4350 ± 0.0071 mg/100g). These findings are consistent with those of Kiani et al. (2021) and Ruiz et al. (2023), who highlighted the effectiveness of mild stress in maintaining pigment retention in resilient genotypes. Top-performing accessions namely ‘CHEN-140’, ‘CHEN-195’, and ‘CHEN-316’ demonstrated stable pigment concentration, reinforcing their suitability for drought-prone environments. Chlorophyll content is a reliable indicator of photosynthetic performance under stress. The highest chlorophyll a and b contents were observed in ‘CHEN-195’, ‘CHEN-316’, and ‘CHEN-140’ under W3, showing strong pigment retention. Lowest values were recorded in ‘PI-614919’, ‘BO-60’, and ‘CICA-17’, indicating weak physiological resilience. Chlorophyll degradation was more pronounced in late stages (90 days), especially in sensitive accessions.

### Principal component analysis

**Figure 7:**
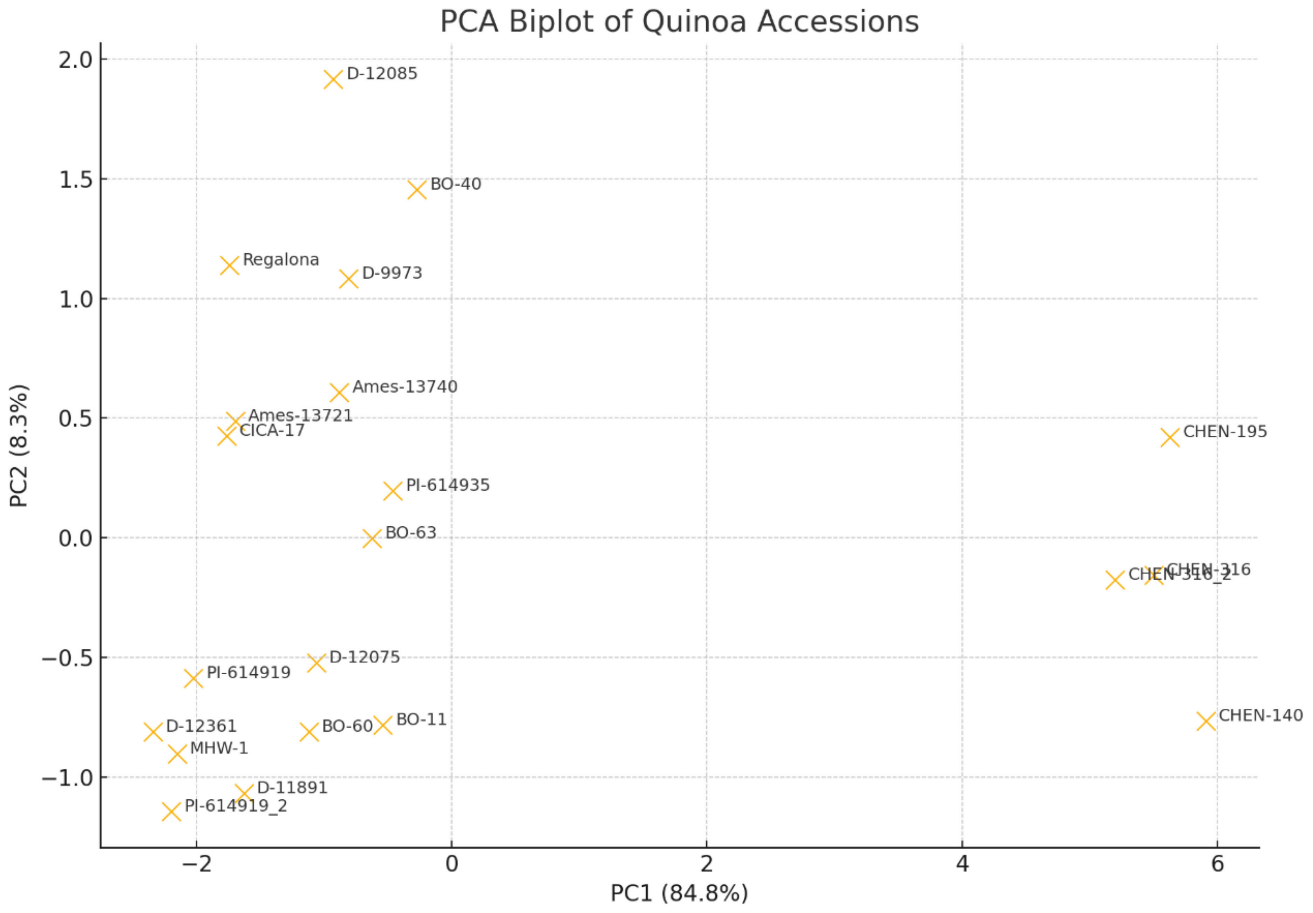
PCA biplot for all 21 quinoa accessions under three water regimes (W1: 15min twice/day), W2: 10min twice/day and W3: 5min twice/day) based on agronomic and yield traits PC1 explains ~49.6% of total variance. PC2 explains ~21.1%, bringing the combined variance explained to about 70.7%, indicating strong dimensionality reduction. Accessions like CHEN-195, CHEN-316, CHEN-140, and BO-40 cluster toward the positive PC1/PC2 quadrant, indicating higher values in traits like yield, height, and drought tolerance index (DTI). Accessions like PI-614919_2, CHEN-140, and D-12361 on the negative PC1 axis may show lower overall trait performance.

Principal component analysis (PCA) of nine agronomic and yield traits revealed clear multivariate differentiation among the 21 quinoa accessions under arid conditions. The first two principal components explained 70.7% of the total variance, with PC1 (49.6%) strongly influenced by seed yield, shoot dry weight, plant height, 1000-seed weight, and drought tolerance index (DTI), indicating that variation in biomass and productivity was the primary source of divergence (**Figure 7**). By contrast, PC2 (21.1%) was mainly associated with chlorophyll content, harvest index, and yield tolerance index (YTI), reflecting differences in physiological efficiency and stress adaptation. This separation suggests that while PC1 captures variation in overall growth vigor and yield capacity, PC2 highlights adaptive mechanisms linked to photosynthetic stability and resource use efficiency under water limitation. The PCA biplot showed broad dispersion of accessions, with CHEN-195, CHEN-140, and CHEN-316 clustered in the high-performing quadrant due to superior yield and biomass traits, while PI-614919_2 and D-12361 grouped on the opposite side with reduced trait values. These findings underscore the utility of PCA in identifying quinoa accessions combining both high yield potential and physiological resilience, supporting previous studies that highlighted PCA as a robust approach for screening stress-adaptive traits in quinoa (Kiani et al., 2021).

### Clustered heatmap analysis

**Figure 8:**
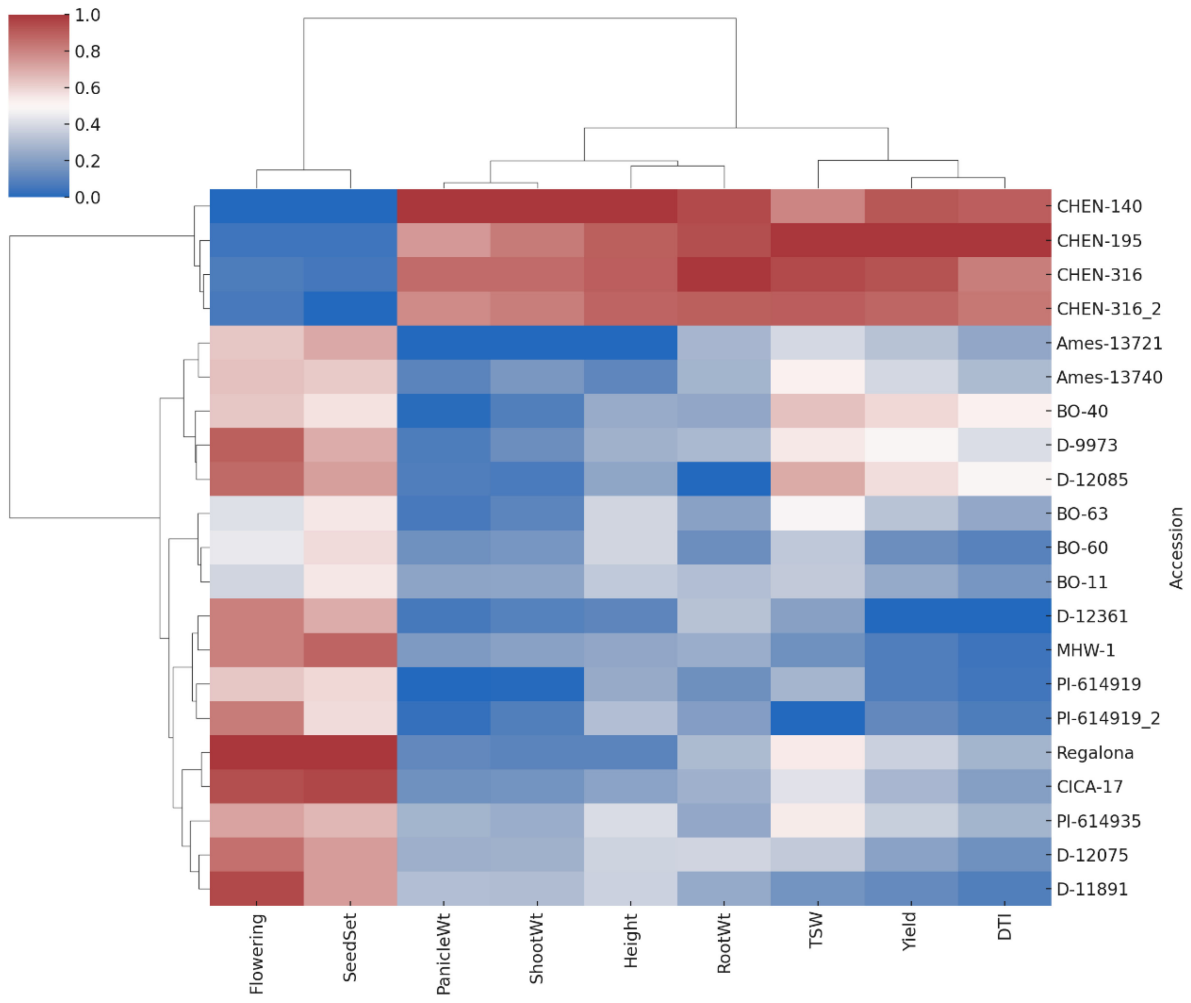
Heatmap with hierarchical clustering of standardized trait values for 21 quinoa accessions across nine agronomic and yield traits. Accessions and traits were clustered using Ward’s method and Euclidean distance. Red shades indicate higher trait values, while blue shades indicate lower values. Clustering reveals groupings of accessions with similar performance profiles under arid conditions.

Hierarchical cluster analysis of the nine agronomic and physiological traits, visualized through a heatmap, further supported the multivariate patterns observed in PCA (**Figure 8**). The heatmap was constructed using z-score normalized trait values, ensuring comparability across traits with different scales. Distinct clusters of quinoa accessions were evident: high-performing accessions such as CHEN-195, CHEN-316, and CHEN-140 grouped together, characterized by elevated biomass, yield, and drought tolerance traits. This “superior performance” cluster likely reflects shared physiological strategies or genetic backgrounds favorable for arid adaptation. In contrast, accessions such as D-12361, PI-614919_2, and Ames-13721 consistently clustered together with comparatively lower trait values. The heatmap also highlighted co-variation among traits; for example, accessions with high shoot and panicle dry weights typically also expressed higher seed yield and DTI values, suggesting functional linkages among growth and yield components under drought stress. Similar clustering patterns were reported by Hirich et al. (2020) and Bazile et al. (2016), who demonstrated the utility of cluster analysis in distinguishing quinoa genotypes by drought-response profiles. Overall, the heatmap complements the PCA results, providing an intuitive visualization of genotype × trait associations that strengthen the identification of promising accessions for breeding in water-limited environments.

## Conclusion

This study evaluated the performance of 21 quinoa accessions under three irrigation regimes (W1: 15 min twice/day, W2: 10 min twice/day, and W3: 5 min twice/day) in arid conditions representative of central Saudi Arabia. The results demonstrated significant genotypic variation and strong genotype × irrigation interactions across phenological, physiological, and yield-related traits. Accessions such as CHEN-195, CHEN-316, and CHEN-140 consistently outperformed others, exhibiting superior chlorophyll stability, biomass accumulation, seed yield, and drought tolerance indices. Their physiological plasticity, delayed senescence, and sustained photosynthetic capacity highlight robust adaptability to both full and deficit irrigation. While W1 generally promoted maximum biomass and yield, some accessions maintained yield stability under moderate (W2) and even limited (W3) irrigation, underscoring their suitability for water-saving cultivation strategies in water-scarce regions. The integration of PCA and clustered heatmap analysis further revealed clear multivariate trait associations, confirming their utility as selection tools for identifying resilient genotypes. These findings have practical implications for breeding and cultivation: CHEN-195, CHEN-316, and CHEN-140 represent promising genetic resources for developing high-yielding, water-efficient quinoa cultivars tailored to arid and semi-arid environments. Their use could enhance food security while reducing irrigation demands in climate-vulnerable regions. While this study revealed substantial variation in quinoa responses to irrigation regimes, several aspects warrant further exploration. The physiological and genetic mechanisms underlying the observed genotype × irrigation interactions remain unclear, and integrating molecular, physiological, and genomic studies would provide deeper insights into the basis of drought resilience. Moreover, although yield tolerance (YTI) and drought tolerance indices (DTI) proved useful in differentiating accessions, their robustness should be validated across broader environments and in conjunction with additional stress indices. Expanding future research to include high-throughput phenotyping, physiological modeling, and marker-assisted or genomic selection will enhance the precision of trait-based breeding. Finally, scaling up promising accessions such as CHEN-195, CHEN-316, and CHEN-140 in multi-environment, farmer-oriented trials will be crucial to confirm their suitability for water-saving strategies and to accelerate the development of resilient, high-yielding quinoa cultivars tailored to arid and semi-arid agro-ecosystems.

## Data Availability

The original contributions presented in the study are included in the article/**Supplementary Material**. Further inquiries can be directed to the corresponding author
