# Supplementary material for: Genotypic variation in yield, physiological traits, and drought tolerance of quinoa (Chenopodium quinoa Willd.) under arid conditions
Source: Front Plant Sci. 2025 Nov 18;16:1679444. doi: 10.3389/fpls.2025.1679444 (PMC12669017; doi:10.3389/fpls.2025.1679444)
Supplement: Supplementary file 1 [file Table1.docx]

**App. Table 1.** ANOVA for the End-Season plant stand/plot of of 21 Quinoa Accessions under Different Irrigation Regimes in arid conditions

| **Source** | **DF** | **SS** | **MS** | **F** | **P** |
| --- | --- | --- | --- | --- | --- |
| Rep | 2 | 35.19 | 17.593 |  |  |
| Water | 2 | 330.49 | 165.243 | 49.14 | 0.0015 |
| Error Rep*Water | 4 | 13.45 | 3.362 |  |  |
| Accession | 20 | 263.53 | 13.177 | 456 | 0.0000 |
| Water*Accessions | 40 | 235.96 | 5.899 | 2.04 | 0.0016 |
| Error Rep*Water*Accessions | 120 | 346.70 | 2.889 |  |  |
| Total | 188 | 1225.31 |  |  |  |

Grand Mean = 19.677 ; CV (Rep*Water) = 9.32; CV (Rep*Water*Accession) = 8.64

**App. Table 2.** ANOVA for Number of days to 50% Flowering of 21 Quinoa Accessions under Different Irrigation Regimes in arid conditions.

| **Source** | **DF** | **SS** | **MS** | **F** | **P** |
| --- | --- | --- | --- | --- | --- |
| Rep | 2 | 1191.41 | 595.70 |  |  |
| Water | 2 | 3218.74 | 1609.37 | 32.71 | 0.0033 |
| Error Rep*Water | 4 | 196.78 | 49.20 |  |  |
| Accession | 20 | 1094.87 | 54.74 | 7.36 | 0.0000 |
| Water*Accessions | 40 | 147.70 | 3.69 | 0.50 | 0.9937 |
| Error Rep*Water*Accessions | 120 | 893.14 | 7.44 |  |  |
| Total | 188 | 6742.65 |  |  |  |

Grand Mean = 37.656; CV (Rep*Water) = 18.63; CV (Rep*Water*Accession) = 7.24

**App. Table 3.** ANOVA for Days to Seed Set of 21 Quinoa Accessions under Different Irrigation Regimes in arid conditions

| **Source** | **DF** | **SS** | **MS** | **F** | **P** |
| --- | --- | --- | --- | --- | --- |
| Rep | 2 | 5060.7 | 2530.33 |  |  |
| Water | 2 | 2842.8 | 1421.40 | 65.84 | 0.0009 |
| Error Rep*Water | 4 | 86.3 | 21.59 |  |  |
| Accession | 20 | 2481.8 | 124.09 | 15.22 | 0.0000 |
| Water*Accessions | 40 | 232.3 | 5.81 | 0.71 | 0.8902 |
| Error Rep*Water*Accessions | 120 | 978.3 | 8.15 |  |  |
| Total | 188 | 116882.3 |  |  |  |

Grand Mean = 51.762; CV (Rep*Water) = 8.98; CV (Rep*Water*Accession) = 5.52

**App. Table 4.** ANOVA for palnt heights of 21 Quinoa Accessions under Different Irrigation Regimes in arid conditions

| **Source** | **DF** | **SS** | **MS** | **F** | **P** |
| --- | --- | --- | --- | --- | --- |
| Rep | 2 | 435.6 | 217.81 |  |  |
| Water | 2 | 13759.8 | 6879.880 | 39.22 | 0.0024 |
| Error Rep*Water | 4 | 701.7 | 175.43 |  |  |
| Accession | 20 | 16529.5 | 826.47 | 26.19 | 0.0000 |
| Water*Accessions | 40 | 3482.9 | 87.07 | 2.76 | 0.0000 |
| Error Rep*Water*Accessions | 120 | 3787.0 | 31.56 |  |  |
| Total | 188 | 38696.6 |  |  |  |

Grand Mean = 88.421; CV (Rep*Water) = 14.98; CV (Rep*Water*Accession) = 6.35

**App. Table 5.** ANOVA for palnt dry weight (g) of 21 Quinoa Accessions under Different Irrigation Regimes in arid conditions

| **Source** | **DF** | **SS** | **MS** | **F** | **P** |
| --- | --- | --- | --- | --- | --- |
| Rep | 2 | 210.9 | 105.46 |  |  |
| Water | 2 | 1487.2 | 743.61 | 5.07 | 0.0800 |
| Error Rep*Water | 4 | 586.8 | 146.69 |  |  |
| Accession | 20 | 28497.4 | 1424.87 | 39.45 | 0.0000 |
| Water*Accessions | 40 | 4586.8 | 114.67 | 3.18 | 0.0000 |
| Error Rep*Water*Accessions | 120 | 4333.9 | 36.12 |  |  |
| Total | 188 | 39703.1 |  |  |  |

Grand Mean = 106.06; CV (Rep*Water) = 11.42; CV (Rep*Water*Accession) = 5.67

**App. Table 6.** ANOVA for root dry weight (g) of 21 Quinoa Accessions under Different Irrigation Regimes in arid conditions

| **Source** | **DF** | **SS** | **MS** | **F** | **P** |
| --- | --- | --- | --- | --- | --- |
| Rep | 2 | 203.84 | 101.919 |  |  |
| Water | 2 | 195.83 | 97.914 | 2.64 | 0.1859 |
| Error Rep*Water | 4 | 148.41 | 37.103 |  |  |
| Accession | 20 | 1716.70 | 85.835 | 24.04 | 0.0000 |
| Water*Accessions | 40 | 300.49 | 7.512 | 2.10 | 0.0011 |
| Error Rep*Water*Accessions | 120 | 428.54 | 3.571 |  |  |
| Total | 188 | 2993.81 |  |  |  |

Grand Mean = 20.231; CV (Rep*Water) = 30.11; CV (Rep*Water*Accession) = 9.34

**App. Table 7.** ANOVA for panicle dry weight (g) of 21 Quinoa Accessions under Different Irrigation Regimes in arid conditions

| **Source** | **DF** | **SS** | **MS** | **F** | **P** |
| --- | --- | --- | --- | --- | --- |
| Rep | 2 | 237.45 | 118.723 |  |  |
| Water | 2 | 631.98 | 315.992 | 10.86 | 0.0242 |
| Error Rep*Water | 4 | 116.36 | 29.090 |  |  |
| Accession | 20 | 6881.41 | 344.070 | 76.58 | 0.0000 |
| Water*Accessions | 40 | 672.54 | 16.813 | 3.74 | 0.0000 |
| Error Rep*Water*Accessions | 120 | 539.13 | 4.493 |  |  |
| Total | 188 | 9078.86 |  |  |  |

Grand Mean = 62.473; CV (Rep*Water) = 8.63; CV (Rep*Water*Accession) = 3.39

**App. Table 8.** ANOVA for shoot dry weight (g) of 21 Quinoa Accessions under Different Irrigation Regimes in arid conditions

| **Source** | **DF** | **SS** | **MS** | **F** | **P** |
| --- | --- | --- | --- | --- | --- |
| Rep | 2 | 80.5 | 40.274 |  |  |
| Water | 2 | 1898.1 | 949.045 | 44.14 | 0.0019 |
| Error Rep*Water | 4 | 86.0 | 21.503 |  |  |
| Accession | 20 | 10511.4 | 515.571 | 76.21 | 0.0000 |
| Water*Accessions | 40 | 995.1 | 24.877 | 3.61 | 0.0000 |
| Error Rep*Water*Accessions | 120 | 827.5 | 6.896 |  |  |
| Total | 188 | 14398.6 |  |  |  |

Grand Mean = 69.296; CV (Rep*Water) = 6.69; CV (Rep*Water*Accession) = 3.79

**App. Table 9.** ANOVA for grain yield/plant(g) of 21 Quinoa Accessions under Different Irrigation Regimes in arid conditions

| **Source** | **DF** | **SS** | **MS** | **F** | **P** |
| --- | --- | --- | --- | --- | --- |
| Rep | 2 | 16.108 | 8.0539 |  |  |
| Water | 2 | 134.277 | 67.1386 | 40.88 | 0.0022 |
| Error Rep*Water | 4 | 6.570 | 1.6424 |  |  |
| Accession | 20 | 703.029 | 35.1515 | 1197.30 | 0.0000 |
| Water*Accessions | 40 | 12.432 | 0.3108 | 10.59 | 0.0000 |
| Error Rep*Water*Accessions | 120 | 3.523 | 0.0294 |  |  |
| Total | 188 | 875.939 |  |  |  |

Grand Mean = 7.6204; CV (Rep*Water) = 16.82; CV (Rep*Water*Accession) = 2.25

**App. Table 10.** ANOVA for reduction (%) in grain yield/plant(g) of 21 Quinoa Accessions under Different Irrigation Regimes in arid conditions

| **Source** | **DF** | **SS** | **MS** | **F** | **P** |
| --- | --- | --- | --- | --- | --- |
| Rep | 2 | 12.9 | 6.43 |  |  |
| Water | 2 | 18043.1 | 9021.53 | 31.90 | 0.0035 |
| Error Rep*Water | 4 | 1131.1 | 282.79 |  |  |
| Accession | 20 | 24.5 | 1.23 | 0.45 | 0.9795 |
| Water*Accessions | 40 | 64.3 | 1.61 | 0.59 | 0.9723 |
| Error Rep*Water*Accessions | 120 | 328.5 | 2.74 |  |  |
| Total | 188 | 19604.4 |  |  |  |

Grand Mean = 11.309; CV (Rep*Water) = 148.69; CV (Rep*Water*Accession) = 14.63

**App. Table 11.** ANOVA for grain yield/plot(g) of 21 Quinoa Accessions under Different Irrigation Regimes in arid conditions

| **Source** | **DF** | **SS** | **MS** | **F** | **P** |
| --- | --- | --- | --- | --- | --- |
| Rep | 2 | 1964 | 982.0 |  |  |
| Water | 2 | 121291 | 60645.6 | 310.32 | 0.0000 |
| Error Rep*Water | 4 | 782 | 195.4 |  |  |
| Accession | 20 | 371546 | 18577.3 | 102.46 | 0.0000 |
| Water*Accessions | 40 | 1014 | 253.6 | 1.40 | 0.0849 |
| Error Rep*Water*Accessions | 120 | 21758 | 181.3 |  |  |
| Total | 188 | 527485 |  |  |  |

Grand Mean = 151.91; CV (Rep*Water) = 9.20; CV (Rep*Water*Accession) = 8.86

**App. Table 12.** ANOVA for reduction(%) in seed yield/plot(g) of 21 Quinoa Accessions under Different Irrigation Regimes in arid conditions

| **Source** | **DF** | **SS** | **MS** | **F** | **P** |
| --- | --- | --- | --- | --- | --- |
| Rep | 2 | 19.5 | 9.7 |  |  |
| Water | 2 | 39424.6 | 19712.3 | 220.14 | 0.0001 |
| Error Rep*Water | 4 | 358.2 | 89.5 |  |  |
| Accession | 20 | 3283.0 | 164.2 | 4.43 | 0.0000 |
| Water*Accessions | 40 | 2977.6 | 74.4 | 2.01 | 0.0200 |
| Error Rep*Water*Accessions | 120 | 4442.7 | 37.0 |  |  |
| Total | 188 | 50505.6 |  |  |  |

Grand Mean = 16.576; CV (Rep*Water) = 57.09; CV (Rep*Water*Accession) = 36.71

**App. Table 13.** ANOVA for seed yield (kg ha⁻¹)g) of 21 Quinoa Accessions under Different Irrigation Regimes in arid conditions

| **Source** | **DF** | **SS** | **MS** | **F** | **P** |
| --- | --- | --- | --- | --- | --- |
| Rep | 2 | 196429 | 98214 |  |  |
| Water | 2 | 1.212E+07 | 6064582 | 310.39 | 0.0000 |
| Error Rep*Water | 4 | 78153.9 | 19538 |  |  |
| Accession | 20 | 3.715E+07 | 1857746 | 102.46 | 0.0000 |
| Water*Accessions | 40 | 1014341 | 15359 | 1.40 | 0.0850 |
| Error Rep*Water*Accessions | 120 | 2175806 | 18132 |  |  |
| Total | 188 | 5.275E+07 |  |  |  |

Grand Mean = 1519.1; CV (Rep*Water) = 9.20; CV (Rep*Water*Accession) = 8.86

**App. Table 14.** ANOVA for reduction (%) for seed yield (kg ha⁻¹)g) of 21 Quinoa Accessions under Different Irrigation Regimes in arid conditions

| **Source** | **DF** | **SS** | **MS** | **F** | **P** |
| --- | --- | --- | --- | --- | --- |
| Rep | 2 | 1946 | 973 |  |  |
| Water | 2 | 3942465 | 1971232 | 220.14 | 0.0001 |
| Error Rep*Water | 4 | 35818 | 8954 |  |  |
| Accession | 20 | 328300 | 16415 | 4.43 | 0.0000 |
| Water*Accessions | 40 | 297755 | 7444 | 2.01 | 0.0020 |
| Error Rep*Water*Accessions | 120 | 444271 | 3702 |  |  |
| Total | 188 | 5050555 |  |  |  |

Grand Mean = 165.76; CV (Rep*Water) = 57.09; CV (Rep*Water*Accession) = 3671

**App. Table 15.** ANOVA for yield tolerance index of 21 Quinoa Accessions under Different Irrigation Regimes in arid conditions

| **Source** | **DF** | **SS** | **MS** | **F** | **P** |
| --- | --- | --- | --- | --- | --- |
| Rep | 2 | 0.0169 | 0.0085 |  |  |
| Water | 2 | 25.5966 | 12.7983 | 556.84 | 0.0000 |
| Error Rep*Water | 4 | 0.0919 | 0.0230 |  |  |
| Accession | 20 | 1.3191 | 0.0660 | 2.73 | 0.0004 |
| Water*Accessions | 40 | 1.7528 | 0.0438 | 1.81 | 0.0074 |
| Error Rep*Water*Accessions | 120 | 2.9034 | 0.0242 |  |  |
| Total | 188 | 31.6808 |  |  |  |

Grand Mean = 0.5100; CV (Rep*Water) = 29.72; CV (Rep*Water*Accession) = 30.50

**App. Table 16.** ANOVA for drought tolerance index of 21 Quinoa Accessions under Different Irrigation Regimes in arid conditions

| **Source** | **DF** | **SS** | **MS** | **F** | **P** |
| --- | --- | --- | --- | --- | --- |
| Rep | 2 | 0.0040 | 0.0020 |  |  |
| Water | 2 | 48.2393 | 24.1196 | 3489.32 | 0.0000 |
| Error Rep*Water | 4 | 0.0276 | 0.0069 |  |  |
| Accession | 20 | 31.8593 | 1.5930 | 70.56 | 0.0000 |
| Water*Accessions | 40 | 16.3408 | 0.4085 | 18.09 | 0.0000 |
| Error Rep*Water*Accessions | 120 | 2.7092 | 0.0226 |  |  |
| Total | 188 | 99.1803 |  |  |  |

Grand Mean = 0.7145; CV (Rep*Water) = 11.64; CV (Rep*Water*Accession) = 21.03

**App. Table 17.** ANOVA for weight of 1000 seeds(g) of 21 Quinoa Accessions under Different Irrigation Regimes in arid conditions

| **Source** | **DF** | **SS** | **MS** | **F** | **P** |
| --- | --- | --- | --- | --- | --- |
| Rep | 2 | 1.2280 | 0.61398 |  |  |
| Water | 2 | 11.3029 | 5.65144 | 52.94 | 0.0013 |
| Error Rep*Water | 4 | 0.4270 | 0.10676 |  |  |
| Accession | 20 | 25.6199 | 1.28100 | 20.53 | 0.0000 |
| Water*Accessions | 40 | 3.2310 | 0.08078 | 1.29 | 0.1444 |
| Error Rep*Water*Accessions | 120 | 7.4867 | 0.06239 |  |  |
| Total | 188 | 49.2955 |  |  |  |

Grand Mean = 2.9061; CV (Rep*Water) = 11.24; CV (Rep*Water*Accession) = 8.59

**App. Table 18.** ANOVA for leaf Cholorophyl a contents after 30 days (mg/100g) of 21 Quinoa Accessions under Different Irrigation Regimes in arid conditions

| **Source** | **DF** | **SS** | **MS** | **F** | **P** |
| --- | --- | --- | --- | --- | --- |
| Rep | 2 | 0.00077 | 0.00038 |  |  |
| Water | 2 | 0.24962 | 0.12581 | 21.12 | 0.0075 |
| Error Rep*Water | 4 | 0.02364 | 0.00591 |  |  |
| Accession | 20 | 1.13423 | 0.02671 | 492.82 | 0.0000 |
| Water*Accessions | 40 | 0.01382 | 0.00035 | 3.00 | 0.0000 |
| Error Rep*Water*Accessions | 120 | 0.01381 | 0.00012 |  |  |
| Total | 188 | 1.43588 |  |  |  |

Grand Mean = 0.5305; CV (Rep*Water) = 14.49; CV (Rep*Water*Accession) = 2.02

**App. Table 19.** ANOVA for leaf Cholorophyl a contents after 60 days (mg/100g) of 21 Quinoa Accessions under Different Irrigation Regimes in arid conditions

| **Source** | **DF** | **SS** | **MS** | **F** | **P** |
| --- | --- | --- | --- | --- | --- |
| Rep | 2 | 0.00102 | 0.00051 |  |  |
| Water | 2 | 0.60531 | 0.15265 | 15.88 | 0.0125 |
| Error Rep*Water | 4 | 0.03845 | 0.00961 |  |  |
| Accession | 20 | 1.12505 | 0.05625 | 598.16 | 0.0000 |
| Water*Accessions | 40 | 0..01345 | 0.00034 | 3.58 | 0.0000 |
| Error Rep*Water*Accessions | 120 | 0.01129 | 0.00009 |  |  |
| Total | 188 | 1.49456 |  |  |  |

Grand Mean = 0.5856; CV (Rep*Water) = 16.74; CV (Rep*Water*Accession) = 1.66

**App. Table 20.** ANOVA for leaf Cholorophyl a contents after 90 days (mg/100g) of 21 Quinoa Accessions under Different Irrigation Regimes in arid conditions

| **Source** | **DF** | **SS** | **MS** | **F** | **P** |
| --- | --- | --- | --- | --- | --- |
| Rep | 2 | 0.00143 | 0.00072 |  |  |
| Water | 2 | 0.36220 | 0.188110 | 15.17 | 0.0136 |
| Error Rep*Water | 4 | 0.04776 | 0.01194 |  |  |
| Accession | 20 | 0.84893 | 0.04245 | 353.48 | 0.0000 |
| Water*Accessions | 40 | 0.01156 | 0.00029 | 2.41 | 0.0001 |
| Error Rep*Water*Accessions | 120 | 0.01441 | 0.00012 |  |  |
| Total | 188 | 1.28660 |  |  |  |

Grand Mean = 0.6360; CV (Rep*Water) = 17.18; CV (Rep*Water*Accession) = 1.72

**App. Table 21.** ANOVA for leaf Cholorophyl b contents after 30 days (mg/100g) of 21 Quinoa Accessions under Different Irrigation Regimes in arid conditions

| **Source** | **DF** | **SS** | **MS** | **F** | **P** |
| --- | --- | --- | --- | --- | --- |
| Rep | 2 | 0.12540 | 0.06270 |  |  |
| Water | 2 | 0.02435 | 0.01218 | 9.85 | 0.0285 |
| Error Rep*Water | 4 | 0.00494 | 0.00124 |  |  |
| Accession | 20 | 0.35750 | 0.01787 | 35.16 | 0.0000 |
| Water*Accessions | 40 | 0.00317 | 0.00008 | 0.16 | 1.0000 |
| Error Rep*Water*Accessions | 120 | 0.06100 | 0.00051 |  |  |
| Total | 188 | 0.57637 |  |  |  |

Grand Mean = 0.3475; CV (Rep*Water) = 10.12; CV (Rep*Water*Accession) = 6.49

**App. Table 22.** ANOVA for leaf Cholorophyl b contents after 60 days (mg/100g) of 21 Quinoa Accessions under Different Irrigation Regimes in arid conditions

| **Source** | **DF** | **SS** | **MS** | **F** | **P** |
| --- | --- | --- | --- | --- | --- |
| Rep | 2 | 0.39889 | 0.19945 |  |  |
| Water | 2 | 0.03541 | 0.01771 | 11.48 | 0.0220 |
| Error Rep*Water | 4 | 0.00617 | 0.00154 |  |  |
| Accession | 20 | 0.39691 | 0.01985 | 41.83 | 0.0000 |
| Water*Accessions | 40 | 0.00460 | 0.00011 | 0.24 | 1.0000 |
| Error Rep*Water*Accessions | 120 | 0.05693 | 0.00047 |  |  |
| Total | 188 | 0.89890 |  |  |  |

Grand Mean = 0.3957; CV (Rep*Water) = 9.93; CV (Rep*Water*Accession) = 5.50

**App. Table 23.** ANOVA for leaf Cholorophyl b contents after 90 days (mg/100g) of 21 Quinoa Accessions under Different Irrigation Regimes in arid conditions

| **Source** | **DF** | **SS** | **MS** | **F** | **P** |
| --- | --- | --- | --- | --- | --- |
| Rep | 2 | 1.03672 | 0.51836 |  |  |
| Water | 2 | 0.04631 | 0.02316 | 9.73 | 0.0291 |
| Error Rep*Water | 4 | 0.00952 | 0.00238 |  |  |
| Accession | 20 | 0.41120 | 0.02056 | 138.35 | 0.0000 |
| Water*Accessions | 40 | 0.00559 | 0.00014 | 0.94 | 0.5776 |
| Error Rep*Water*Accessions | 120 | 0.01783 | 0.00015 |  |  |
| Total | 188 | 1.52717 |  |  |  |

Grand Mean = 0.4467; CV (Rep*Water) = 10.92; CV (Rep*Water*Accession) = 2.73
